# Genome-wide identification and analysis of TCP family genes in *Medicago sativa* reveal their critical roles in Na^+^/K^+^ homeostasis

**DOI:** 10.1186/s12870-023-04318-4

**Published:** 2023-06-06

**Authors:** Mingxiao Zhang, Shangqian Qin, Jianping Yan, Lin Li, Mingzhi Xu, Yanrong Liu, Wanjun Zhang

**Affiliations:** College of Grassland Science and Technology, China Agricultural University, Beijing, 100193 China

**Keywords:** *Medicago sativa*, *MsTCPs*, Salt tolerance, *MIM319*, Na^+^/K^+^ homeostasis

## Abstract

**Background:**

*Medicago sativa* is the most important forage world widely, and is characterized by high quality and large biomass. While abiotic factors such as salt stress can negatively impact the growth and productivity of alfalfa. Maintaining Na^+^/K^+^ homeostasis in the cytoplasm helps reduce cell damage and nutritional deprivation, which increases a salt-tolerance of plant. Teosinte Branched1/ Cycloidea/ Proliferating cell factors (TCP) family genes, a group of plant-specific transcription factors (TFs), involved in regulating plant growth and development and abiotic stresses. Recent studies have shown TCPs control the Na^+^/K^+^ concentration of plants during salt stress. In order to improve alfalfa salt tolerance, it is important to identify alfalfa *TCP* genes and investigate if and how they regulate alfalfa Na^+^/K^+^ homeostasis.

**Results:**

Seventy-one *MsTCPs* including 23 non-redundant *TCP* genes were identified in the database of alfalfa genome (C.V XinJiangDaYe), they were classified into class I PCF (37 members) and class II: CIN (28 members) and CYC/TB1 (9 members). Their distribution on chromosome were unequally. *MsTCPs* belonging to PCF were expressed specifically in different organs without regularity, which belonging to CIN class were mainly expressed in mature leaves. *MsTCPs* belongs to CYC/TB1 clade had the highest expression level at meristem. Cis-elements in the promoter of *MsTCPs* were also predicted, the results indicated that most of the *MsTCPs* will be induced by phytohormone and stress treatments, especially by ABA-related stimulus including salinity stress. We found 20 out of 23 *MsTCPs* were up-regulated in 200 mM NaCl treatment, and *MsTCP3/14/15/18* were significantly induced by 10 μM KCl, a K^+^ deficiency treatment. Fourteen non-redundant *MsTCPs* contained miR319 target site, 11 of them were upregulated in *MIM319* transgenic alfalfa, and among them four (*MsTCP3/4/10A/B*) genes were directly degraded by miR319. *MIM319* transgene alfalfa plants showed a salt sensitive phenotype, which caused by a lower content of potassium in alfalfa at least partly. The expression of potassium transported related genes showed significantly higher expression in *MIM319* plants.

**Conclusions:**

We systematically analyzes the *MsTCP* gene family at a genome-wide level and reported that miR319-*TCPs* model played a function in K^+^ up-taking and/ or transportation especially in salt stress. The study provide valuable information for future study of *TCP* genes in alfalfa and supplies candidate genes for salt-tolerance alfalfa molecular-assisted breeding.

**Supplementary Information:**

The online version contains supplementary material available at 10.1186/s12870-023-04318-4.

## Introduction

Alfalfa is the important forage world widely, and is characterized by high quality, large biomass and strong stress tolerance. While abiotic stresses such as salinity stress can severely affect alfalfa development and production. Thus, it is crucial to breed alfalfa varieties with high abiotic tolerance. In 2020, the genome information of alfalfa was published [1], numerous genes have been identified that may act in response to abiotic stress. Some TFs in alfalfa have been reported responding to salinity, such as Q-type C2H2 zinc-finger protein (*C2H2-ZFP*) [2], *MADS-box* [3], and *SPL* family [4], which provide important genetic resources to breed salinity-resistant alfalfa varieties.

TCP (Teosinte Branched1/ Cycloidea/ Proliferating Cell Factors) gene family was firstly documented in 1999 [5], they are a group of plant-specific genes encoding TFs (transcription factors) with TCP domain. The TCP proteins are characterized with a 59-amino acid basic helix-loop-helix (bHLH) motif, and are considered to be involved in DNA binding, protein–protein interaction and nuclear targeting [6]. According to the amino acid sequences of the TCP domain, TCPs can be divided into two main classes: class I (also known as TCP-P class or PCF class) and class II (or TCP-C) [7]. TCPs belonging to Class II can be further subdivided into two clades, the CIN (CINCINNATA) and the CYC/TB1 (CYCLOIDEA/TEOSINTE BRANCHED 1) subclades [8]. Generally, the class I genes are mostly involved in promoting cell division and differentiation in diverse biological processes ranging from seed germination, leaf and floral organ development and senescence [9–11]. Class II *TCP* genes are mainly related to the development of lateral organs, part of them participate in plant stress resistance. *TCP* members belonging to CYC/TB1 clade mainly involved in regulating floral development, shoot branching and organ development [12–14]. It has been proved that the mRNAs of several CIN *TCPs* could be targeted and degraded by microRNA319 (miR319, one kind of small non-coding RNA) [15–17]. miR319-*TCPs* model is an essential genetic regulator in plants and play vital roles in plant development. Such as overexpressing miR319 or repressing its target *TCPs* both show abnormally wavy rosette leaves and serrated leaves [18], and miR319-*TCP4* has been reported in regulating *LOX2*, which encode a key enzyme in jasmonate (JA) synthesis, and regulates plant leaf senescence [17, 19].

Recently, there has been an increasing interest in the role of the TCP family genes in plant salt stresses adaptation [20, 21]. For class I *TCPs*, over-expression of *OsTCP19* enhanced salt tolerance through regulating ABA signal transduction [22]. For class II *TCP*s, the miR319-*TCPs* model also plays conserved positive roles in *Medicago truncatula*, switchgrass and creeping bentgrass salt tolerance [23–25]. And, it was interested that overexpression miR319 transgenic switchgrass and creeping bentgrass showed higher K^+^ content under normal condition [24, 25]. It also reported that *OsPCF2* potentially activate the expression of *OsNHX1*, a K^+^-Na^+^/H^+^ antiporter gene induced by salinity [26]. Under salt stress condition, decreasing cytoplasmic Na^+^ concentration and increasing K^+^ concentration, a suitable K^+^/Na^+^ ratio in the cytoplasm can be obtained, thus preventing cell damage and nutrient deficiency [27]. However, it is largely unknown that whether, and how, *TCPs* regulate plant K^+^ content.

In this study, we want to give an insight on TCP family genes in alfalfa, and how they response to Na^+^-excess and K^+^-deficiency condition. And, we generated the *MIM319* transgenic alfalfa, verified the miR319-MsTCP pathway could affect the salt tolerance of alfalfa by influence the K^+^ content through physiological experiment of salt stress, and analyzed the possible molecular mechanism.

## Materials and methods

### Identification of the *MsTCPs* in alfalfa

*MsTCPs* protein sequences in alfalfa were obtained from protein annotation file according to *Medicago sativa* Genome Database via InterProScan (v. 5.17–56.0) [28], and were confirmed in the Plant Transcriptional Regulatory Map (PlantRegMap) online (http://plantregmap.gao-lab.org/). The obtained *MsTCPs*’ sequences were applied to SMART (http://smart.embl-heidelberg.de/) to conduct domain analysis to confirm whether belongs to TCP family. The molecular weight (MW) and isoelectric point (PI) of each protein were calculated using ExPASy (http://web.expasy.org).

### *MsTCPs* chromosomal distribution analysis

Information of chromosomal location of *MsTCPs* and the chromosomal length were obtained from tetraploid alfalfa genome database [1], and figure of the distribution of *TCPs* on chromosome was drawn via TBtools.

### TCP Phylogenetic and domain analysis of MsTCP family

TCP protein sequences of *M. sativa* (*MsTCPs*) with *A. thaliana* (*AtTCPs*) [8] and *M. truncatula* (*MtTCPs*) [29] were used to construct an unrooted phylogenetic tree using MEGA5.0 (https://megasoftware.net). DNAMAN was used to conduct sequences alignment of *MsTCPs*. Multiple protein sequences alignment was carried out with Jalview software11 (http://www.jalview.org).

### Gene structure and cis-element analysis of *MsTCP*s

The CDS and corresponding genomic DNA sequences of *MsTCPs* were obtained in the alfalfa genome database. The diagrams of exon, intron and conserved domains of *MsTCPs* genes were generated using TBtools software [28].

The upstream sequences (2 kb) of the *MsTCPs* coding region were retrieved from the alfalfa genomic database and submitted to PlantCARE (http://bioinformatics.psb.ugent.be/webtools/plantcare/html) to identify regulatory elements involved in hormone and stressed responses. Including abscisic acid (ABA)-responsive elements (ABRE), involved in ABA responsiveness; MBS, MYB binding site involved in drought-inducibility; TCA-elements and salicylic acid responsive elements (SARE), involved in salicylic acid responsive; P-box, TATC-box and GARE-motif, involved in gibberellin-responsive element; TGA-element and AuxRR-core, involved in auxin responsive; TGACG-motif and CGTCA-motif, involved in MeJA-responsiveness; low temperature responsive elements (LTR), involved in low-temperature response; and TC-rich repeats, involved in defense and stress response.

### Detection of *MsTCP*s expression patterns in different organs

To detect the expression patterns of *MsTCPs* in different organs, total RNA from apical meristem (MS), young leaves (YL, top leaves), mature leaves (OL, the fourth leave form the top), young stems (YS, the first internode from the top), mature stems (MS, the fourth internode from the top) and root (R, 5 mm root tips) in alfalfa (Cultivar: Zhongmu NO.1) was extracted using Trizol reagent.

### Salt and K^+^ deficiency treatment

Alfalfa plants were propagated via stem-cutting. 7-week-old plants were transferred into 1/4 Hoagland’s solution (containing 1 mM KCl, 0 mM NaCl) for cultivated for 48 h as preculture. After precultured, plants were transferred into 1/4 Hoagland’s solution containing 200 mM NaCl for 12 h as salt treatment, or 1/4 modified Hoagland’s solution containing 10 μM KCl for 24 h as K^+^ deficiency treatment. About ten roots (3 cm length from tip upward) were collected at 0, 1, 3, 6, 12 and 24 h (only K^+^ deficiency treatment) after salt or K^+^ deficiency treatment to extract RNA, each treatment had 3 biological replications.

### Prediction and identification of miR319 targeted *MsTCPs*

miR319 target site prediction was performed using CDS of candidate *MsTCPs* via psRNATarget (http://plantgrn.noble.org/psRNATarget). 5' RLM-RACE was used to validate predicted miR319 cleavage sites in *MsTCPs* experimentally, primers used in this experiment were listed in Table S2. In brief: total RNA was extracted and ligated with the 5’ adaptor ligation RNA (Sangon Biotech, Shanghai, China) by T4 RNA ligase. The ligated product was reverse‐transcribed into the first-strand cDNA using primer complementary to the 5’ adaptor ligation RNA. The cDNA was subsequently PCR‐amplified using GeneRacer 5’primer and MsTCP_GSP_R primer pairs. The PCR products were purified, ligated into the pMD19-T vector and sequenced. Finally, the sequencing results were analyzed to verify the miR319 cleavage site in *MsTCPs* [24].

### Obtain and identification of transgenic alfalfa plants

The miR319 precursor genes of *Arabidopsis*, *Medicago truncatula* and rice were obtained from miRBase database (http://www.mirbase.org/), and were used as templates to blast in genome of alfalfa (https://www.alfalfatoolbox) to obtain *MsMIR319* precursor genes. And the miRBase database was used to predict the mature miR319 sequence produced by *MsMIR319s*. To blocking miR319 expression, we transferred pZh01:*MIM319B* plasmid into alfalfa by *Agrobacterium*-mediated transformation as our previous report [30]. Transgenic alfalfa plants were obtained and identified by stem-loop qRT-PCR.

### Salt treatment of *MIM319* alfalfa

*MIM319* transgenic plants and WT plants were propagated by stem-cutting. Two-month old plants were cultured in 1/4 Hoagland’s solution containing 250 mM NaCl for 3 d. Leaves and roots of WT and transgenic plants were collected respectively to measure the concentration of K^+^ and Na^+^. Briefly, dried samples were grinded, then about 50 mg of powder was taken into a 15 ml glass test tube with cover, 10 ml of deionized water was added in a boiling water bath and extract for 2 h, fix the volume into a 50 ml volumetric flask, filter and then determine the concentration of Na^+^ and K^+^ in the filtrate with flame spectrophotometer. Each treatment had three replications.

### *MIM319* plants under different level of salt stress

Seedlings grown for 4 weeks via stem-cutting were cleaned and transferred to 100 mL brown bottles, containing 100 mL of the following reagents: NaCl concentrations of 0, 140, 160, 180, 200 mM solution (1/4 Hoagland nutrient solution), then were treated for 48 h (photoperiod 14 h light/8 h dark; temperature 25 °C; humidity 50%).

### Prediction of *TCPs* binding sites

According to the annotation file of alfalfa genome, genes related to K^+^ up-taking and transportation were selected. The promoters of these genes were analyzed on JASPAR (https://jaspar.genereg.net/) to predict the presence of TCPs binding site. Genes containing TCP binding sites were named after the blast result on NCBI (https://blast.ncbi.nlm.nih.gov/).

### RNA extraction and expression pattern detection

Total RNA was extracted using Trizol reagent. One microgram of total RNA was reverse transcription into cDNA following the protocol of a reagent kit (Takara RR047 A), the kit can remove the genomics contamination. For miR319 stem-loop qRT-PCR, One microgram of total RNA was reverse transcription using stem-loop PT primer (Table S1). Using cDNA as template, qRT-PCR reactions were performed using Starlighter SYBR Green qPCR Mix (Beijing Qihengxing Biotechnology Co., LTD, FS-Q1002 kit) with a qTOWER^3^G (analytik jena). The calculation of the relative expression levels following 2^−ΔΔCT^ method [20]. *MsActin* was used as an internal control for normalizing. Primers used in qRT-PCR test were listed in Table S1.

### Statistic analysis

All statistical analyses were performed with the IBM SPSS Statistics program (Version 24). Values are presented as the mean ± standard deviation (SD). For multi-group comparison, *P* values were derived from one-way ANOVA (continuous variables). For all comparisons,* P* < 0.05 was considered as statistically significant.

## Results

### Seventy-one *MsTCPs* genes were identified in alfalfa

Seventy-one *MsTCPs* genes which have intact TCP domains were obtained from alfalfa genome (Table 1). The validated *TCP* genes were named from *MsTCP1* to *MsTCP24* based on the phylogenetic relationship with *AtTCPs* and *MtTCPs*, and the lowercase a, b, c, or d were used to distinguish allele genes which located on homologous chromosome (Table 1).

**Table 1:** *TCP* gene family in *Medicago sativa* from the tetraploid alfalfa (Cultivar: XinJiangDaYe) genome database

Name	Gene ID	CDS	Length(aa)	MW(kDa)	Theoretical pI	Name	Gene ID	CDS	Length(aa)	MW(kDa)	Theoretical pI
MsTCP1a	MS.gene074319	960	319	36194.5	9.67	MsTCP11a	MS.gene051472	621	206	22118.8	8.96
MsTCP1b	MS.gene30219	957	318	36085.4	9.67	MsTCP11b	MS.gene029808	621	206	22104.8	8.96
MsTCP2a	MS.gene022063	1125	374	42698.8	6.68	MsTCP11c	MS.gene055897	621	206	22104.8	8.96
MsTCP2b	MS.gene43204	1122	373	42511.6	6.68	MsTCP11d	MS.gene064482	621	206	22076.7	8.96
MsTCP2c	MS.gene92019	1122	373	42567.6	6.68	MsTCP12a	MS.gene95781	1266	421	47569.6	6.43
MsTCP2d	MS.gene44793	1122	373	42527.6	6.68	MsTCP12b	MS.gene070930	1266	421	47569.6	6.43
MsTCP3a	MS.gene060651	903	300	32841.1	7.14	MsTCP13a	MS.gene021927	1056	351	39633.7	8.57
MsTCP3b	MS.gene059738	909	302	33141.5	7.13	MsTCP13b	MS.gene48573	1056	351	39672.8	8.57
MsTCP4a	MS.gene36024	1308	435	47331.5	6.73	MsTCP13c	MS.gene44654	1056	351	39600.7	8.57
MsTCP4b	MS.gene032256	1302	433	47041.1	6.59	MsTCP13d	MS.gene91581	1056	351	39646.7	8.57
MsTCP4c	MS.gene007917	1296	431	46872.0	6.61	MsTCP14a	MS.gene074022	1254	417	44209.6	6.42
MsTCP4d	MS.gene34255	1293	430	46,850.0	6.71	MsTCP14b	MS.gene78508	1254	417	44209.6	6.42
MsTCP5a	MS.gene79398	1155	384	43462.1	7.92	MsTCP14c	MS.gene91902	1248	415	43919.3	6.46
MsTCP5b	MS.gene83823	1149	382	43193.8	8.53	MsTCP14d	MS.gene032839	1251	416	44021.4	6.42
MsTCP5c	MS.gene28232	1146	381	43037.5	7.29	MsTCP15	MS.gene033131	1260	419	45864.2	8.02
MsTCP5d	MS.gene93507	1152	383	43316.9	7.92	MsTCP16a	MS.gene054308	405	134	14873.9	7.76
MsTCP6a	MS.gene044458	750	249	27766.2	9.25	MsTCP16b	MS.gene03512	405	134	14658.8	8.82
MsTCP6b	MS.gene033050	750	249	27718.2	9.35	MsTCP16c	MS.gene80502	495	164	18081.7	7.72
MsTCP6c	MS.gene69207	417	138	15734.0	9.77	MsTCP16d	MS.gene42033	393	130	14327.5	9.46
MsTCP6d	MS.gene063503	930	309	34369.2	9.10	MsTCP18	MS.gene038752	1167	388	44616.5	8.50
MsTCP7a	MS.gene29238	699	232	25498.2	8.04	MsTCP19a	MS.gene006349	981	326	34456.3	4.91
MsTCP7b	MS.gene026187	708	235	25891.6	8.05	MsTCP19b	MS.gene34439	981	326	34413.3	4.84
MsTCP7c	MS.gene91110	699	232	25464.2	8.04	MsTCP19c	MS.gene41379	1023	340	35855.7	4.85
MsTCP7d	MS.gene88588	707	238	26276.0	8.05	MsTCP21a	MS.gene93133	780	259	27781.9	9.51
MsTCP8	MS.gene073917	432	143	16100.5	11.55	MsTCP21b	MS.gene029214	783	260	27872.0	9.72
MsTCP9a	MS.gene019430	996	331	36115.0	9.42	MsTCP21c	MS.gene037360	780	259	27768.9	9.51
MsTCP9b	MS.gene36926	570	189	20950.0	9.55	MsTCP22a	MS.gene00616	1566	521	55216.9	6.32
MsTCP9c	MS.gene37539	996	331	36070.9	9.42	MsTCP22b	MS.gene047202	1566	521	55207.8	6.36
MsTCP9d	MS.gene63692	996	331	36115.0	9.42	MsTCP22c	MS.gene002042	1548	515	54279.4	6.32
MsTCP10Aa	MS.gene54881	990	329	36285.5	5.99	MSTCP22d	MS.gene072060	1563	520	55104.7	6.32
MsTCP10Ab	MS.gene043478	990	329	36285.5	5.99	MsTCP23a	MS.gene054305	360	119	12873.8	9.94
MsTCP10Ac	MS.gene028844	990	329	36229.5	6.03	MsTCP23b	MS.gene80495	324	107	11418.2	10.23
MsTCP10Ad	MS.gene31403	990	329	36,285.5	5.99	MsTCP24a	MS.gene34909	1452	483	53564.9	6.75
MsTCP10Ba	MS.gene006670	1011	336	37829.3	6.21	MsTCP24b	MS.gene023326	1437	478	53008.4	6.75
MsTCP10Bb	MS.gene045512	1011	336	37728.1	6.12	MsTCP24c	MS.gene08299	1458	485	53816.2	6.76
MsTCP10Bc	MS.gene031628	1011	336	37784.1	6.04						

Gene characteristics, including length of CDS (Coding Sequence), length of amino acids, protein molecular weight, and theoretical isoelectric point (pI) were analyzed and listed in Table 1. Based on these data, the length of MsTCP proteins ranged from 107 (*MsTCP23b*) to 521 (*MsTCP22a*), and the molecular weight ranged from 11,418.15 kDa (*MsTCP23b*) to 55,105.21 (*MsTCP22a*). Furthermore, the *MsTCPs* were unevenly located on the chromosomes, as shown in Fig. S1. Most *TCP* genes were located on Chr1 and Chr8, with 6 (*MsTCP7/8/11/19/12/21*) and 5 (*MsTCP6/15/18/4/19*) genes, respectively.

### Phylogenetic analysis and classification of *MsTCPs* in alfalfa

**Fig. 1:**
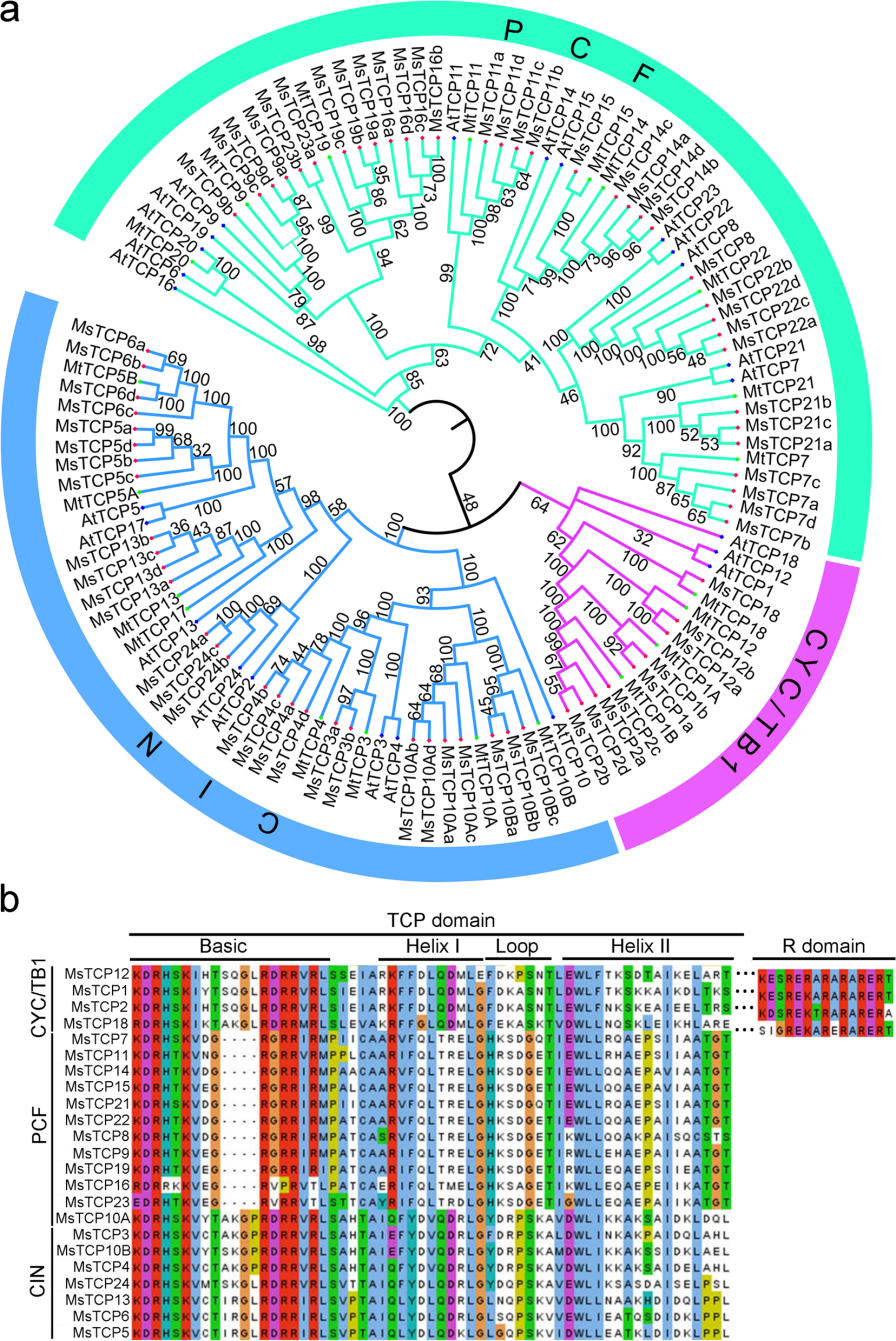
Phylogenetic analysis of TCP proteins and multiple sequence alignment of TCP transcription factors. **a** Phylogenetic analysis of TCP proteins of *A. thaliana* (At)*, M. truncatula* (Mt) and *M.sativa* (Ms)*.* An unrooted neighbor-joining (NJ) tree was constructed using MEGA5.0 (bootstrap value = 1,000). The different colors of branched lines in the subtrees indicate the different TCP subclasses and the corresponding names of subclasses are showed above the arc line. **b** Alignment of TCP domain and R domain of 23 TCP proteins in *M. sativa*. Amino acids that are conserved throughout are shaded in different colors. Conserved domains, including Basic, Helix I, Loop, and Helix II, are shown at the top

In order to elucidate the evolutionary relationship of the *TCPs* among species, complete protein sequence of 71 *MsTCPs*, 24 *AtTCPs,* and 21 *MtTCPs* were used to construct an unrooted phylogenetic tree. The results showed that 71 *MsTCPs* can be divided into two subfamilies, they were referred to as Class I and II according to the classification of *MtTCPs* and *AtTCPs*. Class I (PCF) contained 34 members, and 37 members were classified into Class II which can be further divided into two subclasses: the CIN (28 members) and CYC/TB1 (9 members) (Fig. 1a). Alignment analysis of *MsTCP*s’ protein sequences revealed that all the MsTCP proteins contained a conserved basic helix-loop-helix (bHLH) domain (TCP domain) (Fig. 1b). Only the CYC/TB1 subclass members (*MsTCP12/1/2/18*) include the R domain (Fig. 1b). The results suggested that *MsTCPs* are as evolutionary conservative as other species.

### Gene structure and cis-regulatory elements on *MsTCP*s’ promoters

**Fig. 2:**
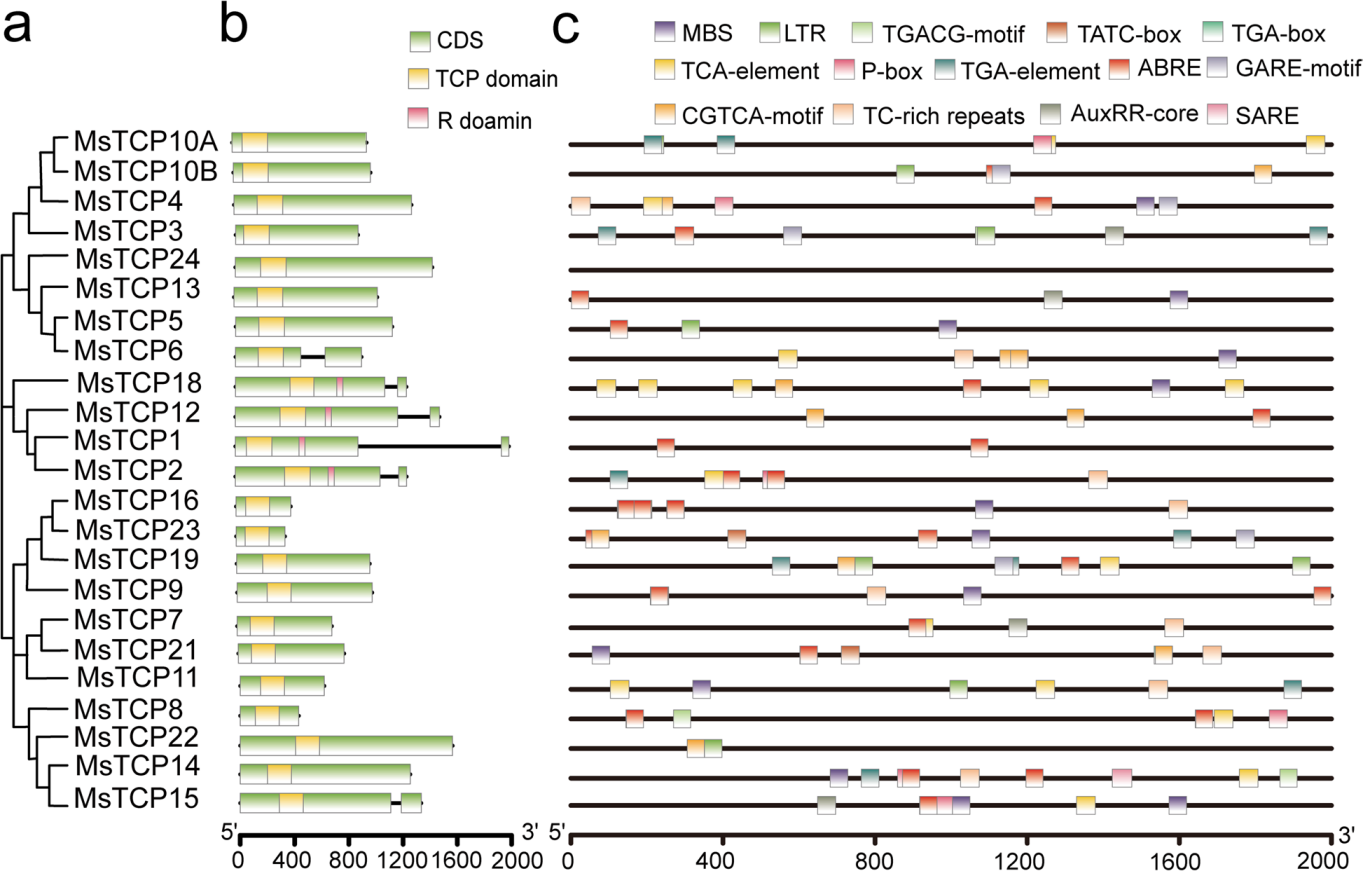
Exon/ intron structure of *MsTCPs* in *M.sativa* and cis-elements analysis of *MsTCPs*’ promoter. **a** Phylogenetic analysis of non-redundant TCP proteins. **b** The exon/ intron organization of *MsTCPs* genes in *M. sativa*. Exons and introns of *MsTCPs* genes were indicated by green rectangles and black lines respectively. The CDS, TCP domain, and R domain are indicated green, yellow, and pink rectangles respectively. The scale was referred to the lengths of the genes. **c** Predicted cis-elements in *MsTCPs*’ promoters. Promoter sequences (− 2000 bp) of *MsTCP* genes are analyzed by PlantCARE. The upstream length to the translation start site can be inferred according to the scale at the bottom

To gain more insight to the evolution of *MsTCPs* gene family on structure, exon/ intron organization of *MsTCPs* genomic DNA and cis-elements on their promoters were analyzed (Fig. 2). Among non-redundant *TCP* genes, 17 out of 23 members had no introns. All the members among CYC/TB1 group contained one intron. Furthermore, *MsTCP*6 and *MsTCP15* contained one intron, respectively (Fig. 2b). Exon/ intron organization within allele genes were also analyzed. Their structures were similar except for *MsTCP6*, *MsTCP9* and *MsTCP16* (Fig. S2). *MsTCP6c/d* had no intron, *MsTCP9b* and *MsTCP16c* had one intron, were different from the others which may due to the evolutionary changed.

Cis-elements related to phytohormone and stress responses on *TCPs*’ promoters were also analyzed (Fig. 2c). The varieties and locations of cis-elements on *TCPs* were manifold, which implied *MsTCPs* functions in multiple metabolic processes. In a total, 95 cis-regulatory elements related to hormones, with 39 elements involved in the abscisic acid response (ABRE), 18 involved in salicylic acid response (17 TCA-elements and 1 SARE), 13 involved in gibberellin response (6 P-box, 2 TATC-box and 5 GARE-motif), 15 involved in auxin response (10 TGA-element,4 auxRR-core and 1 TGA-box) and 15 involved in the MeJA-response (TGACG-motif/ CGTCA-motif). Besides, there were 35 cis-regulatory elements involved in stress response, with 8 involved in low-temperature response (LTR elements), 9 involved in defense and stress response (9 TC-rich repeats) and 13 involved in drought-inducibility (MBS elements). Notably, 19 *TCPs* except *MsTCP10A/11/22/24* contained ABRE (abscisic acid response element) and the total number reached at 39, which suggested that most of them responding to ABA treatment or abiotic stresses. Among the allele genes, obvious differences were observed on their promoters. Except for *MsTCP24*, none of them contained identical numbers or varieties of cis-elements on their promoters (Fig. S3), which implied the evolutionary changes in their promoters are widely existed.

### *MsTCP*s of the same subfamily had similar expression patterns in different organs

**Fig. 3:**
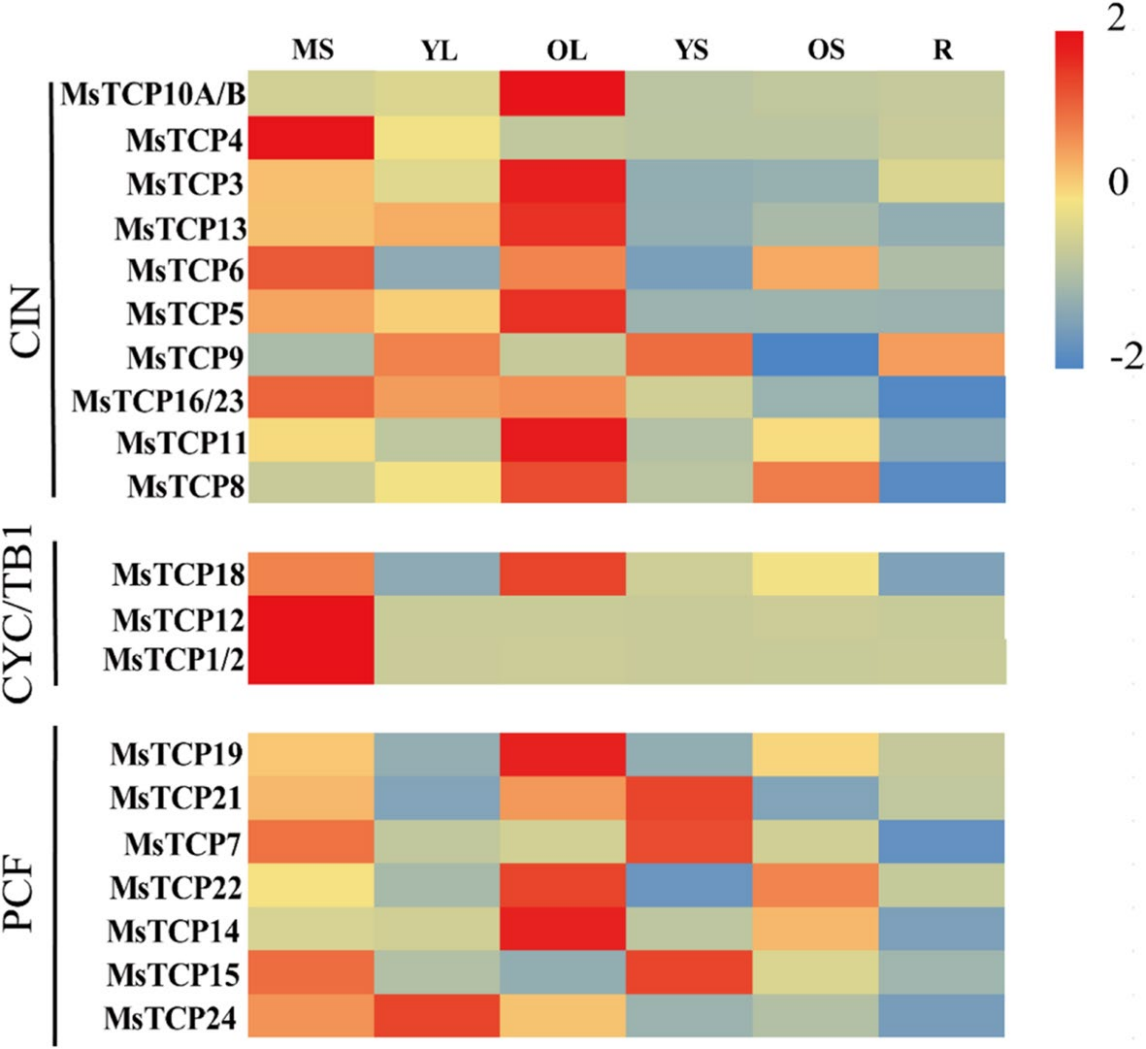
Exon/ intron structure of *MsTCPs* in *M.sativa* and cis-elements analysis of *MsTCPs*’ promoter. **a** Phylogenetic analysis of non-redundant TCP proteins. **b** The exon/ intron organization of *MsTCP* genes in *M. sativa*. Exons and introns of *MsTCP* genes were indicated by green rectangles and black lines respectively. The CDS, TCP domain, and R domain are indicated green, yellow, and pink rectangles respectively. The scale was referred to the lengths of the genes. **c** Predicted cis-elements in *MsTCPs*’ promoters. Promoter sequences (− 2000 bp) of *MsTCP* genes are analyzed by PlantCARE. The upstream length to the translation start site can be inferred according to the scale at the bottom

Expression pattern of *MsTCPs* were detected by qRT-PCR at different organs in alfalfa, including meristem (MS), young leaf (YL), mature leaf (OL), young stem (YS), old stem (OS) and root tip (R) (Table S3). It should be noticed that due to the highly similarity in sequences between *MsTCP1* and *MsTCP2*, *MsTCP16* and *MsTCP23*, *MsTCP10A* and *MsTCP10B*, their expression level cannot be divided through qRT-PCR. As is shown in Fig. 3, each subclasses had their own characteristics in addition to individual genes. *MsTCP*s of CIN clade were predominantly expressed in mature leaves except for *MsTCP23* which mainly expressed in young leaves and young stems, implying these genes may participate in leaf development. *MsTCP4*, *MsTCP16* and *MsTCP9* mainly expressed in meristem. *MsTCP9*, *MsTCP16*, *MsTCP11* and *MsTCP8* presented a relatively low expression level in roots. For CYC/TB1 class *TCPs* predominately expressed at meristems, suggesting they play similar roles in plant developmental processes. Meanwhile, *MsTCP18* also had a relatively higher expression level at mature leaves. The *TCP*s belonging to PCF clade were found expressed specifically in different organs. Such as, *MsTCP19*, *MsTCP22* and *MsTCP14* mainly expressed at mature leaves, while *MsTCP7*, *MsTCP21* and *MsTCP15* showed relatively high expression level at young stems. Apart from those, *MsTCP24* predominantly expressed at young leaves. These results implied that *TCPs* function in multiple plant development processes. However, divergent functions of *MsTCPs* in alfalfa are remaining uncovered, and further studies still to be needed to elucidate specific function on each *MsTCP* gene.

### *MsTCPs* showed different expression pattern after high Na^+^ treatments

**Fig. 4:**
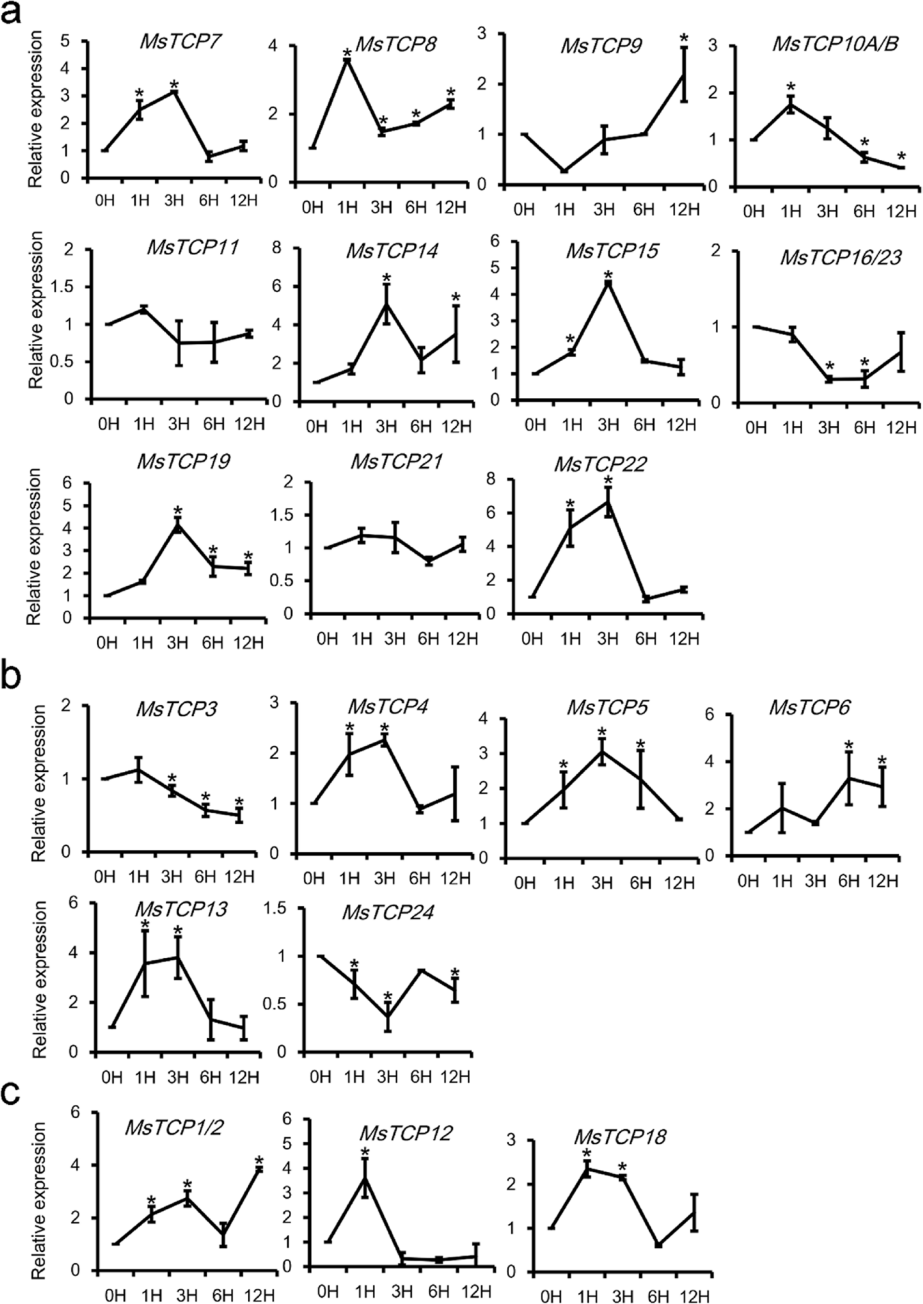
The expression of *MsTCPs* in response to treatment with 200 mM NaCl for 0, 1, 3, 6 and 12 h in roots of alfalfa (7w after cuttage). Data are means with SD for the three replicates. **a** PCF family *TCPs*. **b** CIN family *TCPs*. **c** CYC/TB1 family *TCPs*. Values represent mean ± SD (*n* = 3); asterisks represent significant differences compared to “0 h”, and “*” was considered highly significant *P* < 0.05 (*n* = 3)

Recent study has reported that *TCP*s response to salinity stress [22]. And root is the first organ to feel and response to salinity stress [31]. Thus, to decipher how *MsTCPs* respond to salinity stress, root expression profiles of 23 non-redundant *MsTCPs* under 200 mM NaCl for 0, 1, 3, 6 and 12 h were analyzed. As is shown in Fig. 4, 16 out of 23 *MsTCPs* were up-regulated under 200 mM NaCl treatment at first several hours then down-regulated, and reached their peaks within 3 h, except for *MsTCP9/11/16/23* belonging to PCF family (Fig. 4a), *MsTCP6/24* belonging to CIN family (Fig. 4b), and *MsTCP1/2* of CYC/TB1 subclade (Fig. 4c). *MsTCP1/2* had the same expression pattern as mentioned above, reached the peak after salt treatment for 12 h. *MsTCP9* and *MsTCP6* showed a consistent up-regulated pattern. Besides, the expression level of *MsTCP11* and *MsTCP21* did not change significantly. *MsTCP3*, *MsTCP10A/B*, and *MsTCP24* showed significant decreased after salt treatment for 24 h. Their different expression patterns suggested that they may work at different stages in response to salinity stress.

### Most of *MsTCP* genes response to K^+^ deficiency treatment

**Fig. 5:**
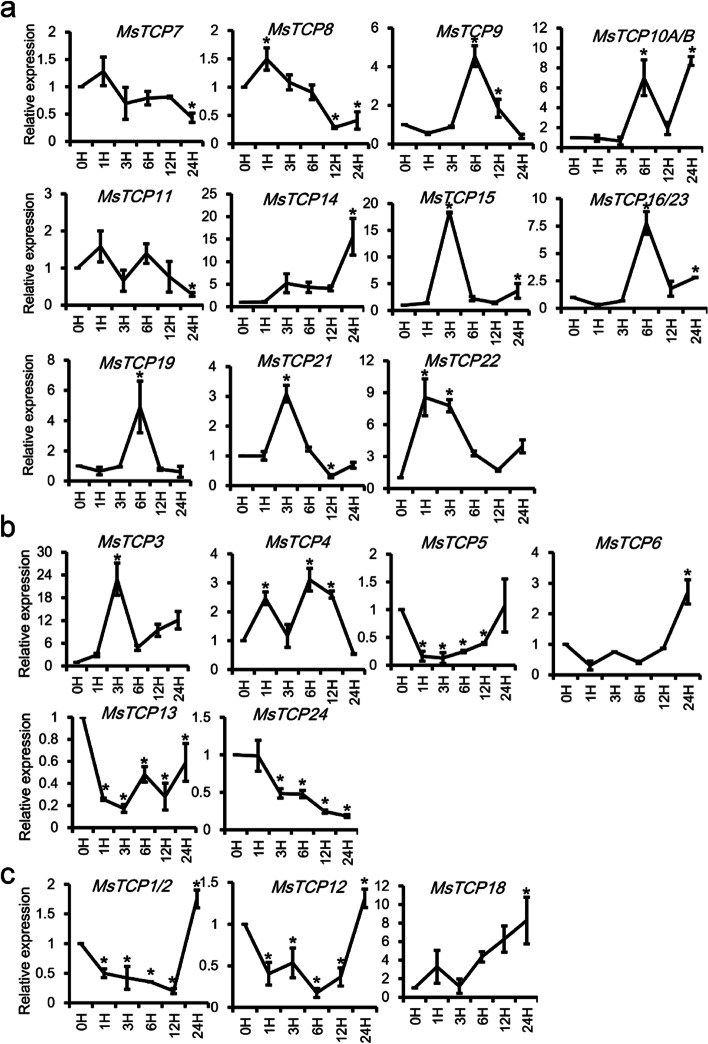
The expression of *MsTCPs* in response to treatment with 200 mM NaCl for 0, 1, 3, 6 and 12 h in roots of alfalfa (7 w after cuttage). Data are means with SD for the three replicates. **a** The expression patterns of PCF family *MsTCPs*, **b** CIN family *MsTCPs*, and **c.** CYC/TB1 family *MsTCPs*. Values represent mean ± SD (*n* = 3); asterisks represent significant differences compared to “0 h”, and “*” was considered highly significant *P* < 0.05 (*n* = 3)

The wild type alfalfa were treated with 10 μM KCl, then the expression pattern of *MsTCPs* in roots was tested by qRT-PCR. As the result shown in Fig. 5, most *MsTCP*s were responded to 10 μM KCl treatment, except for *MsTCP7* and *MsTCP11* remained stable expression and showed no significant change, while expression pattern of the other *MsTCPs* were different. Most of them (*MsTCP3/8/9/15/16/23/19/21/22*) showed an increasing at first several hours then decreasing, and the time they reached their peaks were different (Fig. 5a-c). *MsTCP8, MsTCP15* and *MsTCP4* reached their peaks at 3 h post of treatment, however, *MsTCP9*, *MsTCP16/23* and *MsTCP19* reached the peak in 6 h. *MsTCP10A/B* had the highest expression level at both 6 and 24 h after treated with low K^+^ treatment. *MsTCP14* and *MsTCP6* showed a consistently increasing tendency. Besides, *MsTCP5*, *MsTCP12* and *MsTCP13* decreased firstly then increased after treatment in 3 h. *MsTCP4* reached two peaks at 1 h and 6 h respectively. Besides, only *MsTCP24* remained decreasing under 10 μM KCl treatment. Expression level of *MsTCP3/14/15/18* were increased about 10 times after treatment with 10 μM KCl compared to their expression level before treatment. It should be noticed that *MsTCP15/19/22* had similar expression pattern under salt treatment and K^+^ deficiency situation, while *MsTCP16/23, MsTCP13* and *MsTCP5* showed an opposite expression pattern under NaCl stress and low-concentration of K^+^ treatment, indicating these genes play dominant roles under stresses of high concentration of NaCl and low concentration of K^+^.

### miR319 post-transcriptional cleaveaged *MsTCP3/4/10A/B* and repressed *MsTCP1/2/5/13*

**Fig. 6:**
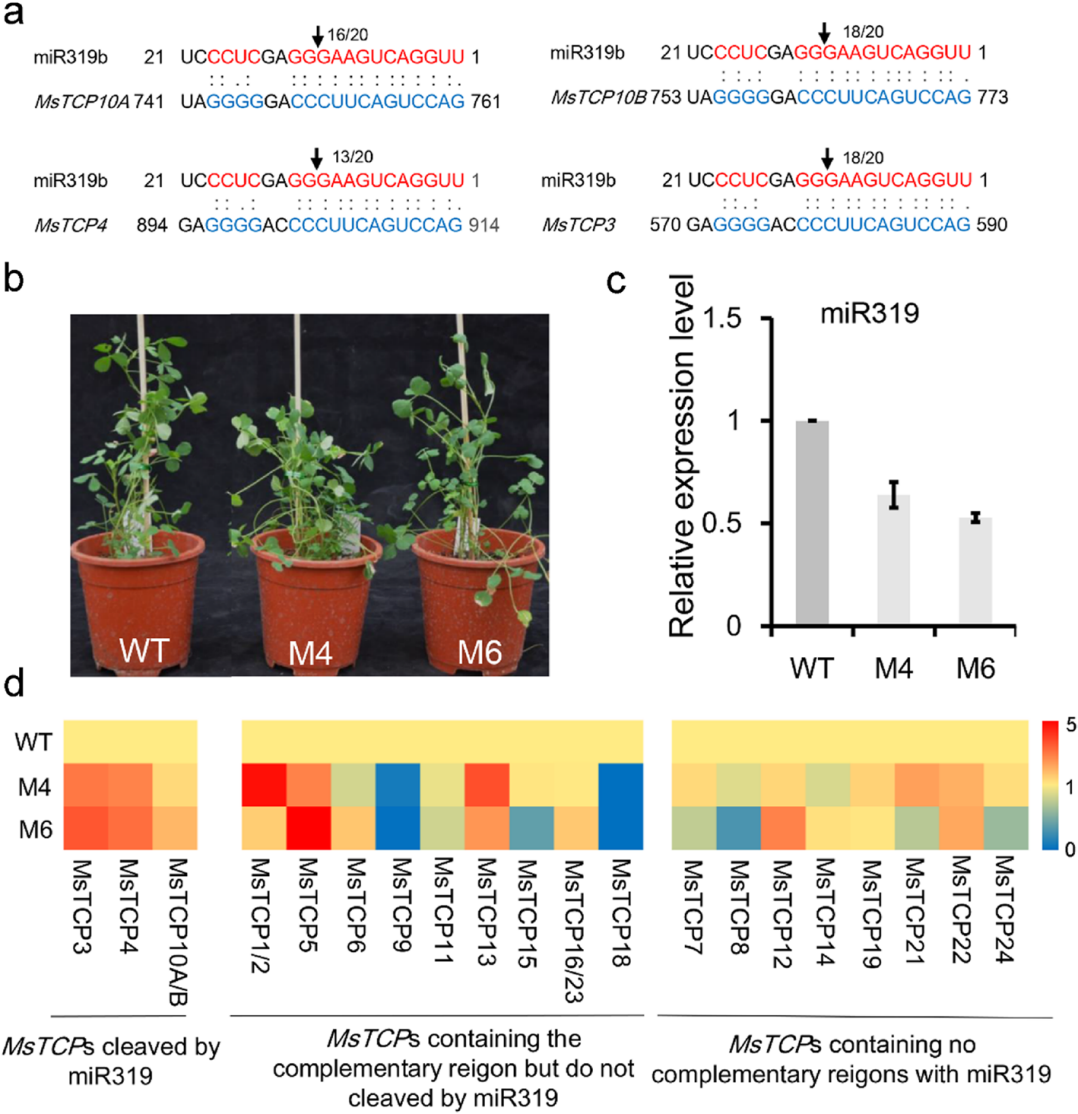
MIR319-target *MsTCPs* identification and the expreession pattern of *MsTCPs* in *MIM319* transgenic plants. **a** Detection of the miR319 cleavage site of the mRNA of *MsTCPs* through 5’ RLM-RACE. Numbers: degradome 5′ ends at arrowhead/total within *MsTCPs* target region (blue). **b** Phenotype of WT plant and *MIM319* plants (M4 and M6). Values represent as mean ± SD (*n* = 3). **c** The expression level of miR319 decreased in *MIM319* plants. **d** The expression level of *MsTCPs* in *MIM319* plants and WT. Values represent the mean of three biological replicates

Suppression of some TCPs by miR319 could be a conserved molecular connection among species [25]. To elucidate this relationship within alfalfa, the supposed *MsMIR319* sequences in alfalfa genome database were selected that were highly homology with *MtMIR319s*, *AtMIR319s* and *OsMIR319s*, and predicted the mature miR319 sequences in miRBase software. We obtained eleven *MsMIR319s* and produced three kind of miR319 sequences (Fig. S4a). Non-redundant *MsTCPs* were searched for the miR319 target sites using psRNATarget, 14 *TCP*s were found containing a miR319 cleavage site. Ten of them belong to PCF class, three belong to CYC/TBI family, and 1 belongs to CIN family (Fig. S4c). 5′ RLM-RACE was then conducted to detect the miR319 cleavage site in vitro. The result showed the mRNAs of *MsTCP10A/B*, *MsTCP3* and *MsTCP4* were directly cleavaged by miR319 between the 10th and 11th bases of miR319 target site with the probabilities of 16/20, 18/20, 13/20 and 18/20, respectively (Fig. 6a). These results suggested that expression of *MsTCP10A/B*, *MsTCP3*, and *MsTCP4* were post-transcriptionally regulated by miR319. To further illuminate the relationship of miR319 and MsTCPs, we overexpressed a *MIM319* gene in alfalfa to blocking in vivo miR319. The stem-loop qRT-PCR results showed that the expression level of miR319 significant decreased in *MIM319* transgenic plants (M4 and M6) compared that in WT (Fig. 6b, c). And the expression level of miR319 cleavaged *MsTCP*s (*MsTCP3*, *4*, *10A/B*) showed a significant increase in Ms than that in WT (Fig. 6d). We also found that the expression level of the other TCPs containing miR319 target site but don’t cleavage were changed in *MIM319* transgenic plants (Fig. 6d). *MsTCP1/2*, *MsTCP5* and *MsTCP13* were up-regulated, while the expression level of *MsTCP9* and *MsTCP18* were decreased, which uncovered that their transcriptional level were regulated by miR319. The expression level of *MsTCPs* without miR319 complementary region showed no significant difference between WT and Ms plants (Table S4).

### Blocking of miR319 decreased alfalfa resistance ability of salt shock due to lower K^+^ content in alfalfa

**Fig. 7:**
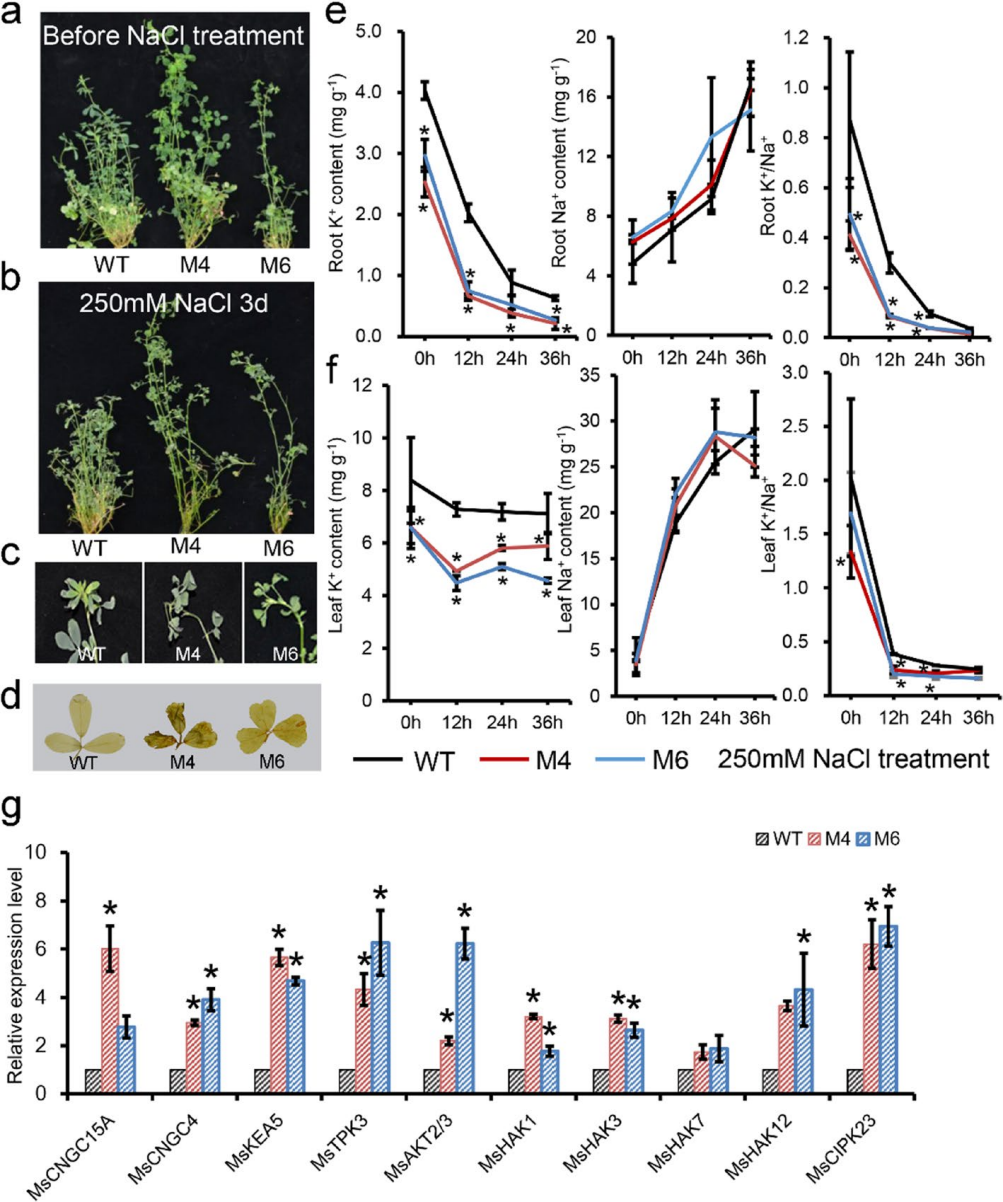
Short-time high-level salt stress in wild-type and *MIM319* transgenic plants (2-month-old plants). **a** Phenotypes of wild-type (WT) and overexpression *MIM319* (M4 and M6) plants before salt stress treatment. **b** Phenotypes of WT and *MIM319* plants after 250 mM NaCl treatment for 3 d. **c** morphology of top leaves of branches after salt treatment. **d** DAB staining of leaves after salt treatment. **e** Concentration of K^+^, Na^+^ and K^+^/Na^+^ ratio of roots at different stage of salt treatment. **f** Concentration of K^+^ and Na^+^, K^+^/Na.^+^ ratio of at top leaves at different stage of salt treatment. Values represent the mean ± SD of three biological replicates, “*”indicate significant differences (*P* < 0.05). **g** The expression pattern of potassium transported related genes in wild-type and *MIM319* transgenic plants under normal condition. Values represent mean ± SD (*n* = 3); asterisks represent significant differences compared with “WT”, and “*” was considered highly significant* P* < 0.05 (*n* = 3)

To test the effects of miR319-*MsTCPs* model on Na^+^/ K^+^ content regulation in alfalfa, four-week old seedlings were used to test the salt tolerance under different level of salt stress. As is shown in Fig. S5, *MIM319* plants showed significant salt sensitivity compared to WT plants. Then, we analyzed the salt shock resistance of WT and Ms by soaking with 250 mM NaCl for 3 d. As is shown in Fig. 7, both WT and Ms alfalfa began to wilt, while the top leaves of WT plants were less damaged compared to those of *MIM319* plants, after treated with 250 mM NaCl for 3 d (Fig. 7a-c). DAB staining assay revealed that more H_2_O_2_ was accumulated in *MIM319* plants than WT plants. The concentration of K^+^ in roots of *MIM319* plants was significantly lower than that in WT plants (*P* < 0.05), and gradually decreased with the prolong of salt treatment hours. Within this process, the concentration of K^+^ in *MIM319* plants remained lower compared to WT plants (Fig. 7e). However, the concentration of K^+^ in WT leaves was stable during salt treatment (*P* < 0.05). Concentration of Na^+^ was gradually increased in both WT and *MIM319* alfalfa, but no significant difference between WT and *MIM319* plants (Fig. 7f). These results indicated that *MIM319* plants reduced salt tolerance in alfalfa by the reduction of K^+^ concentration, which resulted a lower ratio of K^+^/ Na^+^ compared to WT plants.

### K^+^ transport genes were up-regulated in *MIM319* transgenic plants

We detected the expression level of potassium-related iron transport genes which promoters contain TCP binding sites (Table S5). It can be observed that *CNGC*s (Cyclic Nucleotide-gated Channels), HAKs (High-affinity K^+^), and *KEA* (K^+^ efflux anti-porter) were up-regulated, which have been reported to be induced in K^+^ deficiency [32, 33]. *CIPK23* (CBL-Interacting Protein Kinase) was also observed up-regulated in *MIM319* plants, which can directly binds to the promoter of *AKT1* (Arabidopsis K^+^ channel 1), and improve the influx of K^+^ [34]. These results elucidated that *MIM319* showed a salt sensitivity characteristic due to the K^+^ deficiency. However, *AKT2/3* (K^+^ channel 2/3) was induced at in *MIM319* plants, which has been reported to be inhibited in K^+^ free solution [35], which may partly explain the K^+^ deficiency in *MIM319* plants (Fig. 7g).

## Discussion

The TCP transcription factors are widely exist in many monocotyledons and dicotyledons. While the number of them varies among species [8], for example, 23 and 22 *TCP* genes were identified in *A. thaliana* and *O. sativa,* respectively [36]. 21 *MtTCPs* were identified in *M. truncatula* [29], 42 *PvTCPs* were identified in switchgrass [37] and 19 *FvTCPs* were found in strawberry [38]. The genome of ‘Zhongmu No.1’ alfalfa assembled one set of the chromosomes, while the genome of ‘XinJiangDaYe’ assembled the whole four set of chromosomes. Considering that alfalfa is a tetraploid plant with self-incompatibility, there may be differences among genes located at homologous chromosomes, thus the genome sequence of ‘XinJiangDaYe’ was selected to perform the analysis. In alfalfa, 71 *MsTCPs* were identified from the genome of tetraploid (Cultivar: XinJiangDaYe), and there were 23 non-redundant *MsTCP*s. These *TCP*s anchor on chromosomes unevenly, which was also reported in *MtTCP*s [29]. The *MsTCP* gene family were phylogenetically divided into three clades, named as clade PCF, CYC/TB1, and CIN, as that in *A. thaliana* and *M. truncatula* [7, 29], which revealed that *TCPs* in alfalfa was evolutionary conserved. Exon/ intron arrangement of *MsTCPs* also revealed that the genes in the same class/ clade have similar extron/ intron structure.

TCP gene family can influence multiple pathways related to plant growth (such as leaf development, flower morphogenesis phytohormone biosynthesis, and lateral branching) and also evolved in abiotic stress [19, 26, 38, 39]. To predicted *MsTCPs* participate in which phytohormone metabolic pathways, cis-elements in *MsTCPs*’ promoters were analyzed, and hormone response elements and stress response were focused. Intriguingly, most of the *MsTCPs*’ (19 of 23 non-redundant *TCPs*) promoter had at least one abscisic acid responsive element (ABRE), which is responsible for ABA-mediated osmotic stresses signaling [40]. Suggested that abiotic stress such as salinity stress would change the expression level of *MsTCPs*. We also noticed that the cis-elements of allele genes’ promoter changed a lot, which implied the evolutionary changes in the promoters are widely, and resulted in their functional difference.

The expression pattern of *MsTCPs* at organs were analyzed, and different subclasses of *TCPs* have their unique expression pattern. CIN-like clade *TCPs* are involved in regulation of leaf mororphosis, and silencing these genes will lead to an increase of leaf area [18, 41]. Such as *BpTCP7*-overexpressing in *Betula platyphylla* resulted promoted ability of reactive oxygen species scavenging under salinity and drought conditions by integrating multiple hormone metabolic pathways [42]. *TCPs* of CIN clade in alfalfa were also predominantly expressed in mature leaves implying these genes may participate in leaf development. For CYC/TB1 clade, all of them showed high expression level in meristem, which implied their vital functions in floral development and branching process. In chrysanthemums, CYC/TB1 clade *TCPs* were associated with regulation of floral asymmetry [43]. In *Arabidopsis*, this clade genes are destabilized by phytoplasma SAP11 effector, resulting in the proliferation of axillary meristems [44]. Specifically, *AtTCP1* plays an important role in the longitudinal elongation of petioles, rosettes and inflorescence stems [45]. In *M. truncatula*, *MtTCP1A/1B/12* were specifically expressed in flowers, suggesting that they may have similar function. However, the molecular mechanism of these transcription factors on flower development are needed to be further investigated [29]. In cotton and Arabidopsis, both of *TCP12* and *TCP18* (also known as *BRANCHED1 (BRC1)*) are related to branching and axillary bud growth [46, 47], and is also a response factor for spring bud recovery in perennial plants [48], and can directly bind to a *HD-ZIP* gene then improve its transcription level, resulting in enhancing the expression of *NCED3*, and inhibiting bud development [49]. By directly inhibiting the expression of *CsPIN3*, *CsBRC1* inhibit auxin accumulation in axillary buds and inhibit lateral buds growing in cucumber [50]. For CYC/TB1 class *TCPs* in alfalfa predominately expressed at meristem, suggesting they play similar roles in plant developmental processes, as their functions in other species. Compared with other two types of TCP transcription factors, PCF class showed less tissue-/organ-specific expression patterns, and widely expressed in various tissues, suggesting that PCF class members play various regulatory roles at multiple developmental stages in both *Medicago truncula* [29]and *Medicago sativa*.

The expression level of *MsTCPs* in roots after treated with 200 mM NaCl and 10 μM KCl were tested respectively, to elucidate whether *MsTCPs* response to salt stress. We noticed that *MsTCP9*, *MsTCP15* and *MsTCP22* were significantly induced by 200 mM NaCl treatment, besides, *MsTCP3*, *MsTCP14*, *MsTCP15* and *MsTCP18* were significantly induced by K^+^ deficiency. The results implied that these *MsTCPs* may participate in salt stress through K^+^ up-taking or transportation. It was well known that *TCP* genes can be post-transcriptionally regulated by miR319 [25]. Recent research has reported that this miR319-*TCP* model affect multiple development and metabolic pathways. In *A. thaliana*, miR319 affects leaf development and photosynthesis through *TCP*s [15]. Besides it also regulates leaf growth and leaf aging through JA synthesis pathway [17, 51]. MIR319 was also found influence the elongation of internodes, which leads to the decreasing of plant height. Besides, miR319-*TCPs* significantly induced ethylene synthesis and downstream signaling in switchgrass [24]. And under K^+^ deficiency condition, ethylene stimulates the up-regulation of the low potassium ion marker gene *AtHAK5* and improves plant perception of low K^+^ concentration [24].

It has been reported miR319-*TCPs* model functions in salt stress in many species such as *Medicago truncula*, *Panicum virgatum* and *Solanum lycopersicum* [23, 37, 52]. In this study, we identified four *MsTCP* genes (*MsTCP3/4/10A/10B*) can be degraded by miR319, and *MsTCP3* significantly induced by K^+^ deficiency. Which we considered as a candidate gene that will regulate the tolerance of alfalfa via influencing the iron balance. Furthermore, *MIM319* plants were conducted, and it turned out that salt tolerance was reduced in *MIM319* plants compared to wild type alfalfa, which could be caused by the lower content of K^+^ in root and shoot. We also noticed that K^+^-deficiency induced genes were upregulated in *MIM319* compared to wild-type (WT) plants, such as *CNGC*s, *KEA5*, *HAKs* and *CIPK23*. Interesting, a K^+^-efflux channel *MsAKT2/3* was up-regulated in *MIM319* plants, which has been reported as a down-regulated gene under K^+^ deficiency. Which may be part of explanation that salt sensitivity and K^+^ deficiency in *MIM319* plants. Therefore, it is possible that miR319-*MsTCPs* module play a significant role in salt-tolerance by regulating the K^+^ up-taking and transportation pathway.

## Conclusion

In conclusion, we identified 71 (23 non-redundant) *MsTCPs* in tetraploid alfalfa genome, which located on different chromosome and belong to PCF (37 members), CIN (28 members) and CYC/TB1 (9 members) subfamily. And, *MsTCPs* of the same subfamily had similar expression patterns in different organs, but with different expression pattern under Na^+^-excess and K^+^-deficiency situation, suggesting that *MsTCP* genes involved in growth and development regulation and keeping the homeostasis of iron under salt tolerance with function redundancy and specificity. Four MsTCPs (*MsTCP3/4/10A/10B*) were targeted and degraded by miR319 at the post-transcriptional level, and the expression levels of *MsTCP1/2*, *MsTCP5* and *MsTCP13* (containing miR319 target site but do not degraded by miR319) were also up-regulated in *MIM319* plants. *MIM319* plants showed a sensitive to salt stress, and low concentration of K^+^ in roots and leaves, demonstrating that miR319-*TCPs* module involved in the regulation of salt stress via K^+^ up-taking and/ or transportation, at least partly. And, the expression of potassium transported related genes showed higher expression level in *MIM319* transgenic plants than that in WT. The study provide valuable information for future study of *TCP* genes in alfalfa and supplies candidate genes for salt-tolerance alfalfa molecular-assisted breeding.

## Supplementary Information

**Additional file 1: Fig. S1.** Chromosomal distribution of MsTCP genes. **Fig. S2.** Structure analysis of allele TCP genes. **Fig. S3.** Prediction of cis-elements of promoters among allele TCP genes. **Fig. S4.** The sequences of miR319 in alfalfa  and prediction of  miR319-targeted MsTCPs. a. The phylogenetic analysis of *MsMIR319*, *MtMIR319*, *AtMIR319* and *OsMIR319* and their mature miR319 sequences. b. Comparison of *MIM319* sequence with miR319 in alfalfa. c. Prediction of target regions for miR319 in MsTCPs. **Fig. S5.** Comparison of *MIM319* and WT plants under different level of salt stress. **Table S1.** Primers used for qRT-PCR. **Table S2.** Primers used in 5'RLM-RACE. **Table S3.** The expression profiling of *MsTCP* genes in different organs. **Table S4.** The expression level of *MsTCP*s in* MIM319* plants. **Table S5.** Prediction of the binding region of TCP3 and TCP4 on the promoter of potassium-related iron-transport genes.

## Data Availability

All data generated or analyzed during this study are included in this published article and its supplementary information files. The datasets analysed during the current study are available in Medicago Analysis Portal (https://v1.legumefederation.org/data/v2/Medicago/sativa/genomes/).

## Abbreviations

TCP: Teosinte Branched1/ Cycloidea/ Proliferating cell factors
TFs: Transcription factors
CIN: CINCINNATA
CYC/TB1: CYCLOIDEA/TEOSINTE BRANCHED 1
MBS: MYB binding site involved in drought-inducibility
JA: Jasmonate
ABA: Abscisic acid
ABRE: Abscisic acid (ABA)-responsive elements
SARE: Salicylic acid responsive elements
LTR: Low temperature responsive elements
MS: Apical meristem
YL: Young leaves
OL: Mature leaves
YS: Young stems
MS: Mature stems
R: Root tip
PCR: Polymerase Chain Reaction
qRT-PCR: Quantitative Real-time Polymerase Chain Reaction
CNGCs: Cyclic Nucleotide-gated Channels
HAKs: High-affinity K ^+^
KEA: K^+^ efflux anti-porter
CIPK23: CBL-Interacting Protein Kinase 23
AKT: Arabidopsis K^+^ channel
CDS: Coding sequence
aa: Amino acids
kDa: Kilo Dalton
pI: Protein isoelectric point
