# Supplementary material for: Genome-wide identification and analysis of TCP family genes in Medicago sativa reveal their critical roles in Na+/K+ homeostasis
Source: BMC Plant Biol. 2023 Jun 6;23:301. doi: 10.1186/s12870-023-04318-4 (PMC10242995; doi:10.1186/s12870-023-04318-4)
Supplement: Supplementary file 1 — Additional file 1: Fig. S1. Chromosomal distribution of MsTCP genes. Fig. S2. Structure analysis of allele TCP genes. Fig. S3. Prediction of cis-elements of promoters among allele TCP genes. Fig. S4. The sequences of miR319 in alfalfa and prediction of miR319-targeted MsTCPs. a. The phylogenetic analysis of MsMIR319, MtMIR319, AtMIR319 and OsMIR319 and their mature miR319 sequences. b. Comparison of MIM319 sequence with miR319 in alfalfa. c. Prediction of target regions for miR319 in MsTCPs. Fig. S5. Comparison of MIM319 and WT plants under different level of salt stress. Table S1. Primers used for qRT-PCR. Table S2. Primers used in 5'RLM-RACE. Table S3. The expression profiling of MsTCP genes in different organs. Table S4. The expression level of MsTCPs in MIM319 plants. Table S5. Prediction of the binding region of TCP3 and TCP4 on the promoter of potassium-related iron-transport genes. [file 12870_2023_4318_MOESM1_ESM.docx]

Additional files

# Additional Figures


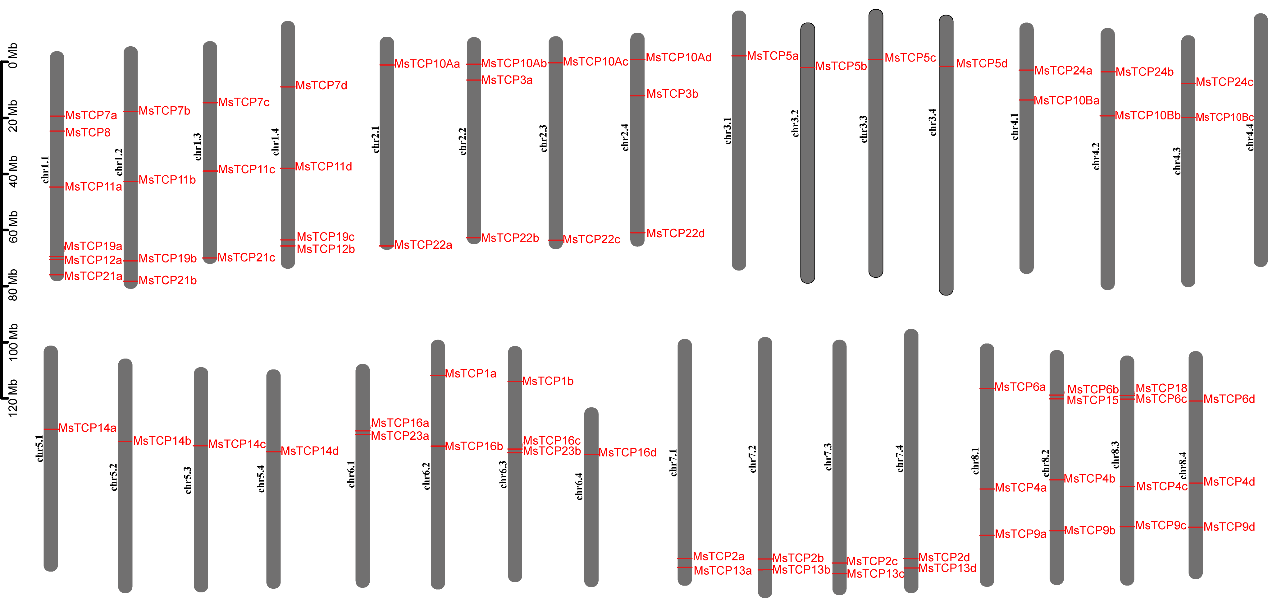


**Fig. S1.** Chromosomal distribution of *MsTCP* genes.

**
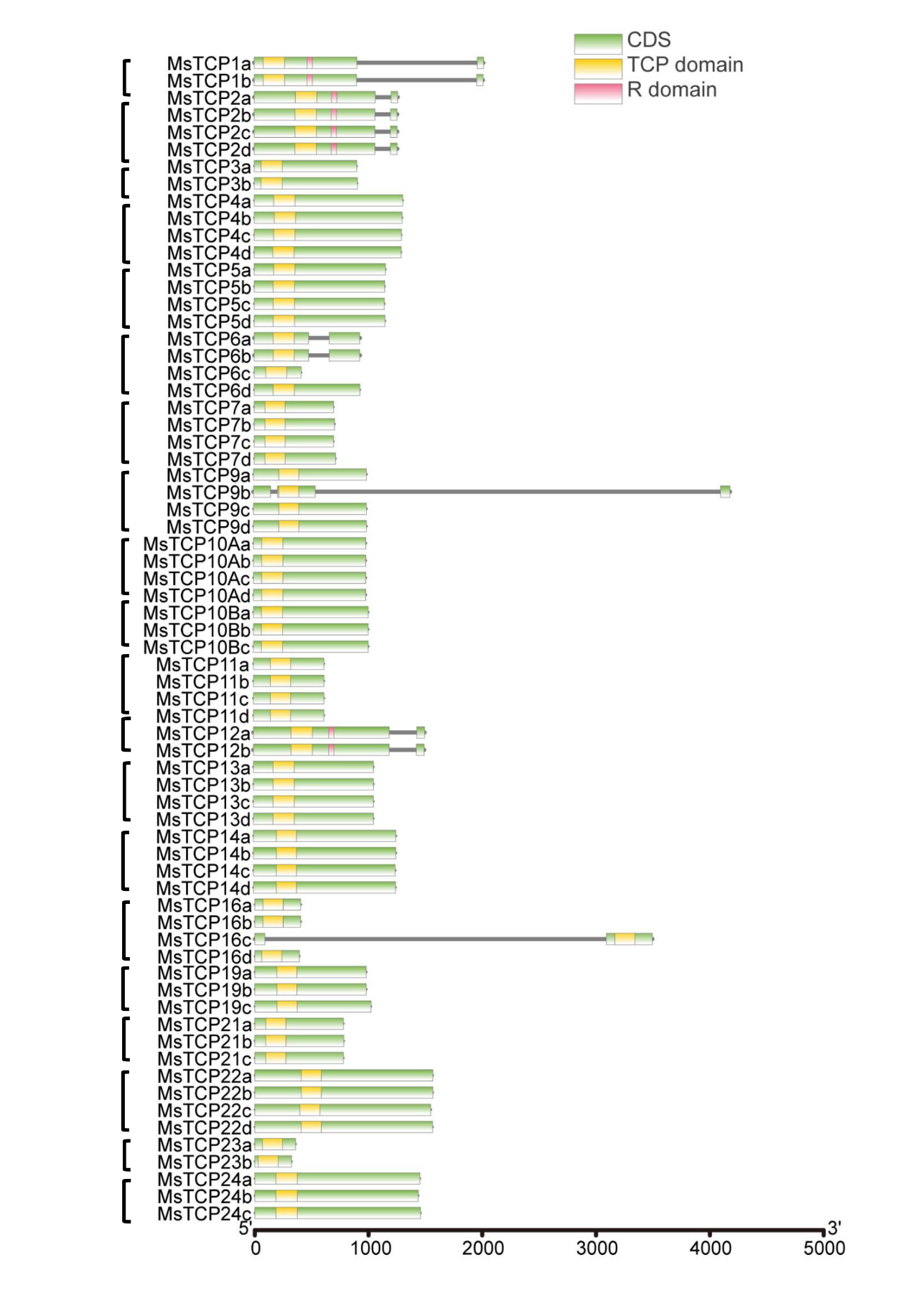
Fig. S2.** Structure analysis of allele *TCP* genes


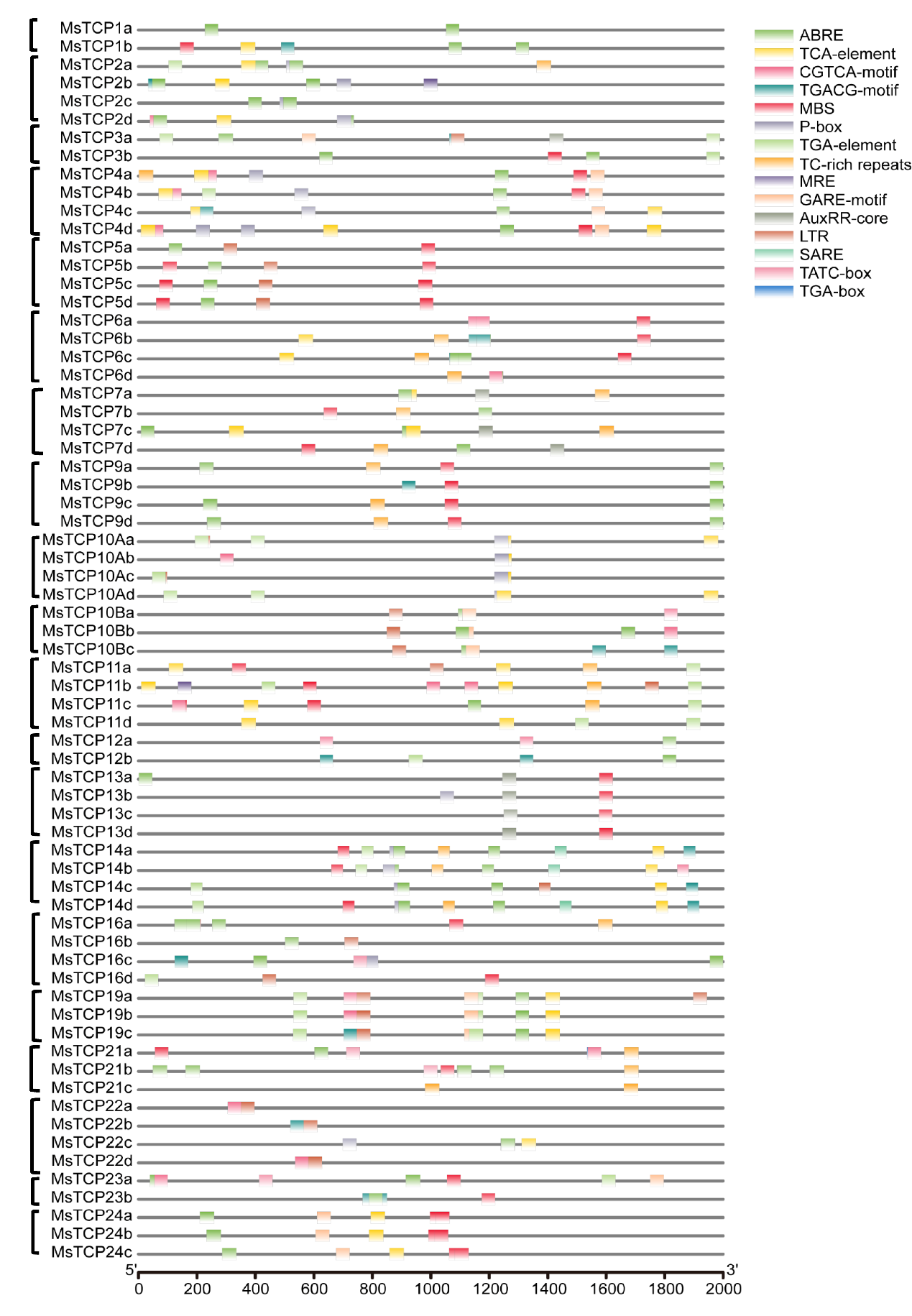


**Fig. S3.** Prediction of cis-elements of promoters among allele *TCP* genes.

**
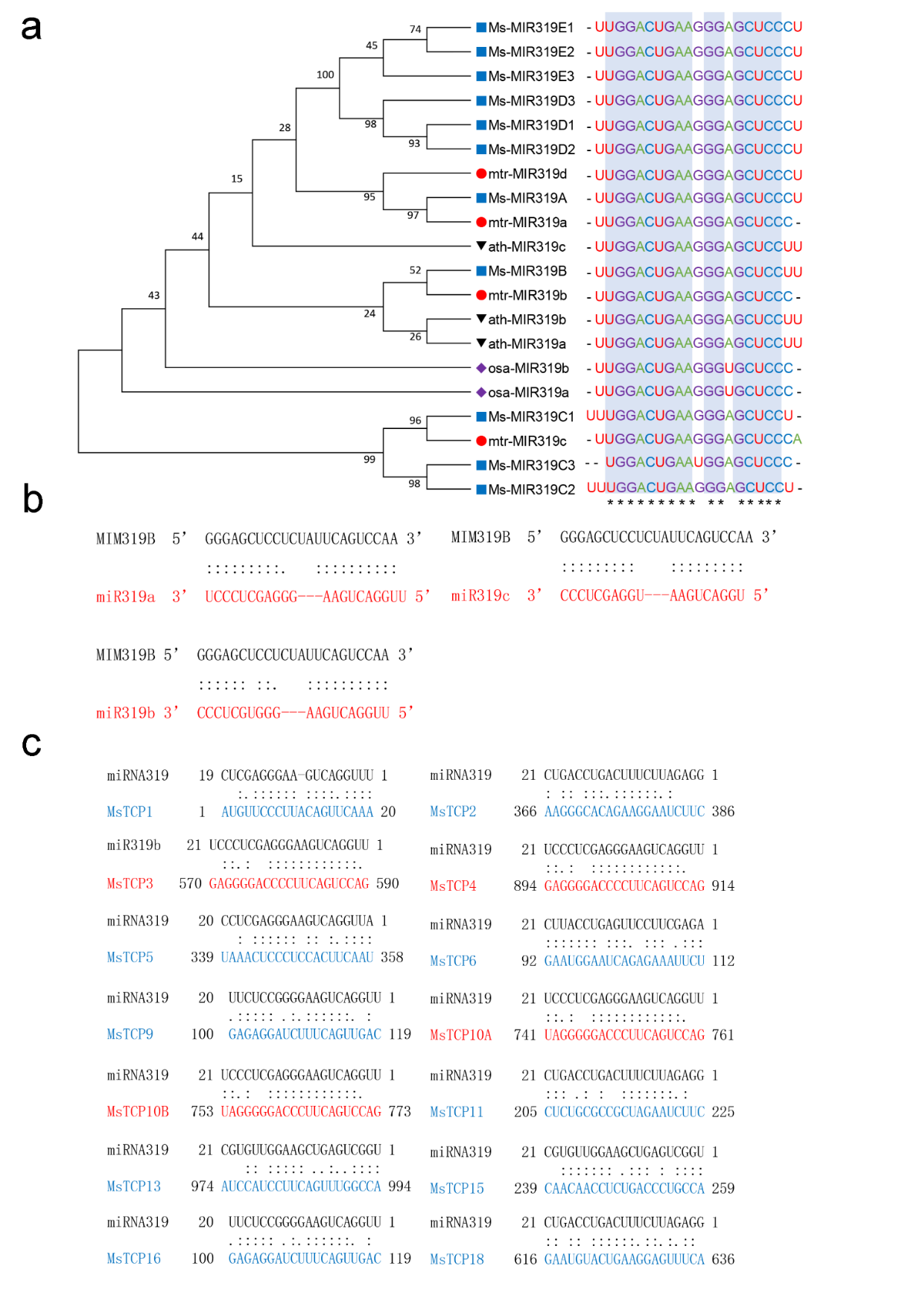
**

**Fig. S4**. The sequences of miR319 in alfalfa and prediction of miR319-targeted *MsTCPs*. **a.** The phylogenetic analysis of *MsMIR319*, *MtMIR319*, *AtMIR319* and *OsMIR319* and their mature miR319 sequences. **b.** Comparison of MIM319 sequence with miR319 in alfalfa. **c.** Prediction of target regions for miR319 in *MsTCP*s

Note:Ms respresents *Medicago sativa*; mtr represents *Medicago truncatula*; ath represents *Arabidopsis thaliana* ; osa represents *Oryza sativa.*

**
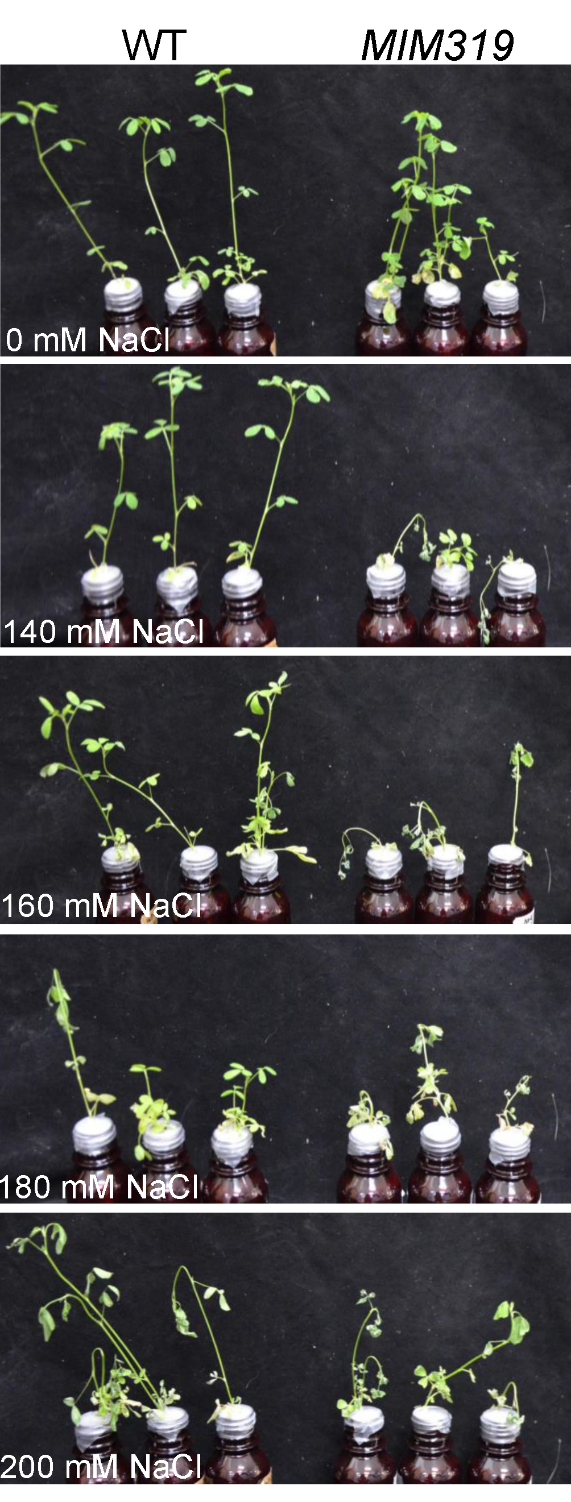
**

**Fig. S5** Comparison of *MIM319* and WT plants under different level of salt stress.

# Additional Tables

**Table S1.** Primers used for qRT-PCR

| Primer Name | Sequence(5'→3') | Primer Name | Sequence(5'→3') |
| --- | --- | --- | --- |
| MsActin_F | CAAAAGATGGCAGATGCTGAGGAT | MsTCP18_F | ATCAGCGGCCTTCGAACTCAAC |
| MsActin_R | CATGACACCAGTATGACGAGGTCG | MsTCP18_R | TTGCTGTGTCGGTCTCTCTTGC |
| MsTCP1/2_F | AGGAAGGTGCAGGAAGTAAACC | MsTCP19_F | AGTGGCGGCGGCGAAGAG |
| MsTCP1/2_R | TCCCTTGCTCTTGCTCTTGC | MsTCP19_R | AAGATTCTGGCGGCGCATGTC |
| MsTCP3_F | AAGGGCCGAGAGACAGACGTG | MsTCP21_F | CAGCAGCAACAGCAACAACAGC |
| MsTCP3_R | AAGGGCCGAGAGACAGACGTG | MsTCP21_R | TCCACCAGACAACGAAGCAAGC |
| MsTCP4_F | CCACCATGACAACCACCACCAC | MsTCP22_F | AGGCAACGCTGCTGCTGTAAC |
| MsTCP4_R | CTGCTGCTGCTCAGACCACAAG | MsTCP22_R | CAACACCACCACCACCACCAG |
| MsTCP5_F | CCTCCGGTTAGACCTTTTCCG | MsTCP24_F | CCCTTCAGTCCAATTCACCGTCTC |
| MsTCP5_R | ACGAGCACTATCATCATCCGGT | MsTCP24_R | GAAGAAGAAGCAGAAGCACCTCCTC |
| MsTCP6_F | CTAGTGGCAGCAGTGGTATGC | miR319_F | CGGCGGTTGGACTGAAGGGT |
| MsTCP6_R | GGCAATGGCATAACAGAAGGACA | miR319_R | GTGCAGGGTCCGAGGT |
| MsTCP7_F | AGACGGAAGAGGAAGACGCA | MsCIPK23_F | GCTCGCAGTGGGAGGTTGAAAG |
| MsTCP7_R | GACGGTTCAGCTTGACGGAG | MsCIPK23_R | AGGCTTTAGGTCTCTGTGGCAAAC |
| MsTCP8_F | TCTTCAACTTCAACCGCCGTCAC | MsCNGC15A_F | TTGCTCCTTCATCTCGTGTTTCTGG |
| MsTCP8_R | CCGTCAACTTTTGTGTGCCTGTC | MsCNGC15A_R | GCCCAAATGAGTACCTGAGGAAGTG |
| MsTCP9_F | CACGCGGAGCCAGCTATCATTG | MsCNGC4_F | GCCACCACAACAACAGAACAAGAC |
| MsTCP9_R | TTGGTGAGGTGGTTGGGATTTTGAG | MsCNGC4_R | GCTGCTCTTCTTCCTCGTCATCATC |
| MsTCP10A/B_F | TGCGTTTCTAGGCCAAGGTTCG | MsKEA5_F | GCTGTTCTTGGAGGGCTGCTTC |
| MsTCP10A/B_R | TCCACCGAGGCATCAGCAATTG | MsKEA5_R | CACCACTGCTGTTGATGACATTGAC |
| MsTCP11_F | CGGTGCCGTCGGTTTCTTGTC | MsTPK3_F | TAGGATGAAGGTGGCATTGGCATTG |
| MsTCP11_R | CGCCGTGCCGTAATCCATCC | MsTPK3_R | CACCATACCCAACCGTCGTAACAG |
| MsTCP12_F | AGCAGAAACAACCAGCAGCA | MsAKT2/3_F | ACATGCTCTTCAACCTTGGCCTAAC |
| MsTCP12_R | ACCCTTCTGTCTCTCAAACCCT | MsAKT2/3_R | ATGGTGCGACGAGTTCCTTCAAC |
| MsTCP13_F | AGGCTTTCAGTACCAACAGCTA | MsHAK12_F | CGCTGTGTTGCTAGGTACGGTTAC |
| MsTCP13_R | GCTGCATTGAGCAACCAATCA | MsHAK12_R | TCCAACTCCAACGCAGCTTCTAAC |
| MsTCP14_F | GCAGTGGAGGAGGAGGAGGTG | MsHAK7_F | GTGATGACGACGACCACTGTCTTAG |
| MsTCP14_R | GCCGTGATGCTGTCCCGATG | MsHAK7_R | GCCACGCTCCACAGAACAGATG |
| MsTCP15_F | GGCCGCCGCATCCGAATG | MsHAK3_F | GCAAGTCAGGCTGTCATCTCTTCG |
| MsTCP15_R | TTCCGGTGGCAGCTATAACAGC | MsHAK3_R | GTCTGACCATTGAGCCACCTTCTG |
| MsTCP16/23_F | TCCGAAGAGTCACACTGTCAACAAC | MsHAK1_F | GTTTCCTTCATGTTTGCCCCAGTTG |
| MsTCP16/23_R | CGGTGGCTTTAATGATTGCAGGTTC | MsHAK1_R | TGCACGATATACTTGTCGGTTCCAC |
| Stem-loop RT primer | GTCGTATCCAGTGCAGGGTCCGAGGTATTCGCACTGGATACGACGGGAGC |  |  |

**Table S2.** Primers used in 5'RLM-RACE.

| Primer Name | Sequence 5'-3' |
| --- | --- |
| GeneRacer 5′ primer | AGGACACTGACATGGACTGAAGGAGTAG |
| MsTCP1_GSP_R | TCTTTGTGAGGAAACAGGAAATACC |
| MsTCP2_GSP_R | AAGTTGGATCTTCCAGTGACAGC |
| MsTCP3_GSP_R | AACGGTGAGAATCAGAGGAAGCAGA |
| MsTCP4_GSP_R | ATACGCCGTCGTGTTCCTCTT |
| MsTCP5_GSP_R | CCTTTAGTCCTTGAATCTATGTCCC |
| MsTCP6_GSP_R | AACTTTGAGGAATATCAAGTGGTGG |
| MsTCP9_GSP_R | CCTTCCTCTGACACGACTATGGC |
| MsTCP10A/B_GSP_R | GCTTGGGGAATTTGTTGTTGAG |
| MsTCP11_GSP_R | GAAACCGACGGCACCGAGGAA |
| MsTCP13_GSP_R | CTTGTTCTTTATTGGATGAATTTTC |
| MsTCP15_GSP_R | GTATAGTTCCAGTTCCGGTGGC |
| MsTCP16_GSP_R | CCTTCCTCTGACACGACTATGGC |
| MsTCP18_GSP_R | AAGGGGTTCCAAATGAGGTTAGT |

**Table S3.** The expression profiling of *MsTCP* genes in different organs.

| Gene | | | *MsTCP1/2* | *MsTCP3* | *MsTCP4* | *MsTCP5* | *MsTCP6* |
| --- | --- | --- | --- | --- | --- | --- | --- |
| **Organ** | meristem | MS | 0.1786±0.0179 | 2.3302±0.1087 | 4.4215±0.0345 | 0.6289±0.0583 | 0.0056±0.0006 |
|  | young leaf | YL | 0.0021±0.0001 | 1.3048±0.1033 | 1.0238±0.1100 | 0.4626±0.0134 | 0.0019±0.0007 |
|  | old leaf | OL | 0.0031±0.0018 | 5.0144±0.1818 | 0.3176±0.0591 | 1.1038±0.0451 | 0.0049±0.0007 |
|  | young stem | YS | 0.0004±0.0000 | 0.0569±0.0197 | 0.2330±0.0168 | 0.0060±0.0022 | 0.0016±0.0007 |
|  | old stem | OS | 0.0000±0.0000 | 0.1450±0.0005 | 0.2334±0.0102 | 0.0121±0.0003 | 0.0044±0.0017 |
|  | root tip | R | 0.0014±0.0001 | 1.2312±0.0368 | 0.4282±0.0277 | 0.0023±0.0002 | 0.0024±0.0009 |
| Gene | | | *MsTCP7* | *MsTCP8* | *MsTCP9* | *MsTCP10A/B* | *MsTCP11* |
| **Organ** | meristem | MS | 17.4806±2.5713 | 3.0343±0.1877 | 0.0016±0.0004 | 2.7309±0.3425 | 0.3635±0.1227 |
|  | young leaf | YL | 9.4558±0.1161 | 4.3207±1.3738 | 0.0034±0.0032 | 3.6856±0.4971 | 0.1550±0.0076 |
|  | old leaf | OL | 10.2514±1.4455 | 8.9140±0.6983 | 0.0018±0.0007 | 27.7269±6.3390 | 0.9738±0.0374 |
|  | young stem | YS | 19.4421±4.9479 | 2.6263±0.6747 | 0.0036±0.0014 | 0.4835±0.0468 | 0.1242±0.0454 |
|  | old stem | OS | 10.1846±0.8294 | 7.4066±2.1124 | 0.0007±0.0001 | 1.1270±0.1395 | 0.3587±0.0073 |
|  | root tip | R | 5.2532±0.3790 | 0.0199±0.0211 | 0.0031±0.0016 | 1.3817±0.0601 | 0.0011±0.0008 |
| Gene | | | *MsTCP12* | *MsTCP13* | *MsTCP14* | *MsTCP15* | *MsTCP16/23* |
| **Organ** | meristem | MS | 0.0568±0.0035 | 2.3550±0.2595 | 4.4215±0.0238 | 5.2319±0.0555 | 0.4086±0.0480 |
|  | young leaf | YL | 0.0008±0.0003 | 2.7140±0.0619 | 1.0238±0.0044 | 2.2042±0.0270 | 0.3260±0.0838 |
|  | old leaf | OL | 0.0007±0.0004 | 4.9081±0.0036 | 0.3176±0.0034 | 1.7212±0.1329 | 0.3451±0.0919 |
|  | young stem | YS | 0.0005±0.0002 | 0.0535±0.0092 | 0.2330±0.0003 | 5.9229±0.3027 | 0.1688±00699 |
|  | old stem | OS | 0.0011±0.0002 | 0.3874±0.0046 | 0.2334±0.0003 | 2.8428±0.1072 | 0.0908±0.0040 |
|  | root tip | R | 0.0003±0.0002 | 0.0141±0.0026 | 0.4282±0.0002 | 1.8935±0.1436 | 0.0014±0.0000 |
| Gene | | | *MsTCP18* | *MsTCP19* | *MsTCP21* | *MsTCP22* | *MsTCP24* |
| **Organ** | meristem | MS | 2.2364±0.0906 | 2.4478±0.2881 | 4.9758±0.5286 | 2.8627±0.2261 | 21.8500±5.1977 |
|  | young leaf | YL | 0.4899±0.1238 | 0.6095±0.0093 | 2.3498±0.0795 | 1.6381±0.0222 | 30.9391±2.5749 |
|  | old leaf | OL | 2.8366±0.2102 | 4.8822±0.2379 | 5.4820±0.0299 | 5.2923±0.1843 | 16.9014±0.4142 |
|  | young stem | YS | 1.0341±0.0980 | 0.5766±0.0295 | 6.9272±0.0291 | 0.8216±0.1516 | 3.4443±0.1013 |
|  | old stem | OS | 1.3610±0.0176 | 2.2166±0.1437 | 2.3447±0.2340 | 4.2714±0.6031 | 5.8900±0.9485 |
|  | root tip | R | 0.3775±0.0249 | 1.2839±0.0045 | 3.3270±0.1547 | 2.0823±0.3091 | 0.0013±0.0003 |

**Table S4.** The expression level of *MsTCPs* in *MIM319* plants.

|  | *MsTCP3* | *MsTCP4* | *MsTCP10A/B* | *MsTCP1/2* | *MsTCP5* |
| --- | --- | --- | --- | --- | --- |
| WT | 1.00±0.00 | 1.00±0.00 | 1.00±0.00 | 1.00±0.00 | 1.00±0.00 |
| M4 | 3.00±0.48 | 2.82±0.37 | 1.31±0.09 | 4.81±0.07 | 2.83±0.11 |
| M6 | 3.60±0.49 | 3.18±0.67 | 1.91±0.09 | 1.54±0.27 | 5.08±0.27 |
|  | *MsTCP6* | *MsTCP9* | *MsTCP11* | *MsTCP13* | *MsTCP15* |
| WT | 1.00±0.00 | 1.00±0.00 | 1.00±0.00 | 1.00±0.00 | 1.00±0.00 |
| M4 | 0.83±0.03 | 0.09±0.00 | 0.92±0.06 | 3.72±0.24 | 1.11±0.10 |
| M6 | 1.65±0.25 | 0.01±0.00 | 0.81±0.00 | 2.49±0.29 | 0.38±0.01 |
|  | *MsTCP16/23* | *MsTCP18* | *MsTCP7* | *MsTCP8* | *MsTCP12* |
| WT | 1.00±0.00 | 1.00±0.00 | 1.00±0.00 | 1.00±0.00 | 1.00±0.00 |
| M4 | 1.06±0.14 | 0.00±0.00 | 1.32±0.11 | 0.87±0.04 | 1.38±0.24 |
| M6 | 1.61±0.24 | 0.00±0.00 | 0.75±0.13 | 0.29±0.00 | 2.83±0.63 |
|  | *MsTCP14* | *MsTCP19* | *MsTCP21* | *MsTCP22* | *MsTCP24* |
| WT | 1.00±0.00 | 1.00±0.00 | 1.00±0.00 | 1.00±0.00 | 1.00±0.00 |
| M4 | 0.85±0.02 | 1.45±0.10 | 2.31±0.73 | 2.03±0.25 | 1.26±0.10 |
| M6 | 1.20±0.72 | 1.10±0.11 | 0.72±0.18 | 2.18±0.26 | 0.61±0.02 |

**Table S5.** Prediction of the binding region of *TCP3* and *TCP4* on the promoter of potassium-related iron-transport genes.

| **Gene name** | **Gene id** | **Annotation** | **Name** | **Start** | **End** | **Strand** | **Predicted sequence** |
| --- | --- | --- | --- | --- | --- | --- | --- |
| AKT2/3 | MS.gene072035 | Arabidopsis K^+^ transport, AKT | MA1035.1.TCP4 | 118 | 125 | - | TGGTCCAC |
|  |  |  | MA1035.1.TCP4 | 119 | 126 | + | TGGACCAA |
|  |  |  | MA1289.1.TCP3 | 116 | 128 | - | TCTTGGTCCACAT |
| TPK3 | MS.gene023125 | Two-pore domain potassium channels,TPK | MA1035.1.TCP4 | 616 | 623 | - | GGGACCAA |
|  |  |  | MA1289.1.TCP3 | 614 | 626 | - | ATAGGGACCAAAA |
|  |  |  | MA1035.1.TCP4 | 1117 | 1124 | - | TGGACAAC |
| CNGC15A | MS.gene75014 | cyclic nucleotide-gated ion channel-like protein, CNGC | MA1035.1.TCP4 | 909 | 916 | + | TGGACCAA |
|  |  |  | MA1035.1.TCP4 | 1558 | 1565 | - | AGGAGCAC |
| CNGC4 | MS.gene070835 |  | MA1289.1.TCP3 | 68 | 80 | + | TTTGGTACCACAT |
|  |  |  | MA1035.1.TCP4 | 71 | 78 | + | GGTACCAC |
| HAK12 | MS.gene038598 | potassium transporter-like protein | MA1035.1.TCP4 | 734 | 741 | + | TGGACCAC |
|  |  |  | MA1035.1.TCP4 | 961 | 968 | - | GGGACCTC |
|  |  |  | MA1035.1.TCP4 | 709 | 716 | + | GGAACCAC |
|  |  |  | MA1289.1.TCP3 | 731 | 743 | + | CCCTGGACCACAT |
|  |  |  | MA1289.1.TCP3 | 959 | 971 | - | TACGGGACCTCTT |
|  |  |  | MA1289.1.TCP3 | 706 | 718 | + | CATGGAACCACGT |
| HAK7 | MS.gene002201 |  | MA1035.1.TCP4 | 1178 | 1185 | + | TGGTCCAC |
|  |  |  | MA1035.1.TCP4 | 1177 | 1184 | - | TGGACCAA |
|  |  |  | MA1289.1.TCP3 | 1175 | 1187 | + | TATTGGTCCACAC |
|  |  |  | MA1289.1.TCP3 | 1175 | 1187 | - | GTGTGGACCAATA |
| HAK3 | MS.gene06028 |  | MA1289.1.TCP3 | 1630 | 1642 | - | TTTGGGTCCATTT |
|  |  |  | MA1035.1.TCP4 | 1035 | 1042 | + | TGGATCAC |
| HAK1 | MS.gene54170 |  | MA1035.1.TCP4 | 536 | 543 | + | TGGATCAC |
| KEA5 | MS.gene30487 | potassium efflux antiporter, KEA | MA1035.1.TCP4 | 107 | 114 | - | GTGACCAC |
|  |  |  | MA1289.1.TCP3 | 105 | 117 | - | CATGTGACCACAA |
|  |  |  | MA1035.1.TCP4 | 293 | 300 | - | TGGAACAC |
| MsCIPK23 | MS.gene97056 | CBL-interacting kinase 23, CIPK23 | MA1035.1.TCP4 | 171 | 178 | - | AGAACCAC |
|  |  |  | MA1035.1.TCP4 | 642 | 649 | - | TGGAGCAC |
